# Characterization of proteome-size scaling by integrative omics reveals mechanisms of proliferation control in cancer

**DOI:** 10.1126/sciadv.add0636

**Published:** 2023-01-25

**Authors:** Ian Jones, Lucas Dent, Tomoaki Higo, Theodoros Roumeliotis, Maria Arias Garcia, Hansa Shree, Jyoti Choudhary, Malin Pedersen, Chris Bakal

**Affiliations:** Chester Beatty Laboratories, Institute of Cancer Research, London SW3 6JB, UK.

## Abstract

Almost all living cells maintain size uniformity through successive divisions. Proteins that over and underscale with size can act as rheostats, which regulate cell cycle progression. Using a multiomic strategy, we leveraged the heterogeneity of melanoma cell lines to identify peptides, transcripts, and phosphorylation events that differentially scale with cell size. Subscaling proteins are enriched in regulators of the DNA damage response and cell cycle progression, whereas super-scaling proteins included regulators of the cytoskeleton, extracellular matrix, and inflammatory response. Mathematical modeling suggested that decoupling growth and proliferative signaling may facilitate cell cycle entry over senescence in large cells when mitogenic signaling is decreased. Regression analysis reveals that up-regulation of TP53 or CDKN1A/p21CIP1 is characteristic of proliferative cancer cells with senescent-like sizes/proteomes. This study provides one of the first demonstrations of size-scaling phenomena in cancer and how morphology influences the chemistry of the cell.

## Teaser

Molecules that super- and subscale with cancer cell size and the consequences for cell growth are defined.

## INTRODUCTION

Eukaryotic cells vary widely in size; there is a billion-fold difference in cell volume between *Xenopus* oocytes [~1-mm diameter, (*1*)=] and phytoplankton (~1 μm) (*2*). As cell size directly affects nutrient acquisition and consumption, diffusive processes, and intracellular protein concentrations, this results in a spectrum of biology (*3*, *4*).

Although notable differences in size are observed when comparing between different cell types, size distributions within proliferating cell types show only modest variance or size “uniformity” (*5*) [coefficients of variation (CVs) typically 0.1 to 0.3] (*6*). The size homogeneity of proliferating cell populations implies the existence of size checkpoints during proliferation, which act to coordinate cell cycle progression and the acquisition of cell mass (*3*, *5*). To maintain a stable size distribution across a population, a checkpoint system can measure the size of individual cells with molecular “rulers” (*7*). Measurements are then coupled to the speed of the division cycle and the acquisition of mass. Such a system would “penalize” cells that deviate from the target volume (a “sizer” system), accelerating or diminishing the cell proliferation rate.

Other mechanisms of size determination have been documented that do not inherently depend on cell size measurements, such as the “adder” or “timer” models where a fixed amount of cell mass is added per cycle (*3*, *8*). However, recent studies allude to similarity between sizer and adder/timer systems, with modest errors in sizer function leading to adder-like behavior (*9*).

Several studies have identified molecular mechanisms of how size measurements are coupled to proliferation and/or growth. In budding yeast, a type of ruler appears to consist of a mechanism where the concentration of a cell cycle progression inhibitor, *Whi5*, becomes diluted with respect to the activator, *Cln3*, as cells grow larger, allowing cell cycle progression only at a critical size (*7*, *10*). The set point appears to be, in part, determined by the concentration of *Whi5* relative to the number of DNA binding sites for the cell cycle activator *SBF* (*11*).

Recently, it has been demonstrated that RB1 (an ortholog of *Whi5*) may have a role in mammalian size control. *RB1* concentration subscales with size across the cell cycle, meaning that in smaller newly born daughters, the activity of *RB1* may exceed that of its agonist, cyclin D1 (*CCND1*). *CCND1* scales with size, and thus as cells grow, there is a point at which the activity of *CCND1* exceeds that of *RB1*, and the cell commits to proliferation (*12*). In normal cells, *CCND1* levels are a function of mitogen signaling and translational activity (*13*). Thus, in normal mammalian cells, cells meet the *RB1:CCDN1* set point for proliferation, by synthesizing *CCND1* while simultaneously diluting *RB1* (*12*).

It is becoming clear that regulation of protein function by diluting or concentrating with cell size is not a rare phenomenon. Many proteins have been shown to “super” or “subscale” (mass fraction increases/decreases) with cell size beyond a small set of proliferative regulators. Recent studies point to histones (*14*, *15*), translational components (*16*), and several metabolic elements (*14*, *17*, *18*) sub/super-scaling with cell size. Not all these proteins will act as size rulers and may instead influence their activity. For example, chromatin-associated histones have been shown to regulate equal partitioning of *Whi5* in asymmetric cell divisions in budding yeast (*19*). Dilution of cell proteins (and intracellular DNA) through excessive growth has also recently been associated with the onset of cell senescence (*17*).

In other cells, size control is highly influenced by cell geometry. For example, in fission yeast (*20*), size is thought to be determined primarily at G_2_-M through the accumulation of localized *CDR2* nodes at the growing mid body, activating cyclin-dependent kinase 1 (*CDK1*) by inhibiting *Wee1* (*21*–*23*). Because *CDR2* accumulation scales with surface area, this provides a means by which the detection of cell geometry influences a size checkpoint. Other work has shown analogous regulation of size by surface area or volume in bacteria (*24*). Different geometric quantities, such as surface area, may serve to mediate size control in different cell types through coupling to signaling proteins.

Because most studies on size control use either yeast, bacteria, or normal mammalian cells, there is little understanding of size determination in cancer. Classic studies indicate that increased size and morphological heterogeneity are histological measures of cancer grade, with large and more morphologically varied cancers, tending to be more pathogenic (*25*). Only highly heterogeneously sized lines induced tumors upon transplantation in mice (*26*). This diversification of cell size has been shown to be cell autonomous and not an artifact of the environment (*26*). Together, these observations suggest modification of cell size in malignant tissues and that this contributes to (or coincides with) increased cellular fitness. However, the exact relationship between size and disease is poorly understood.

Consistent with the idea that size and size heterogeneity are associated with oncogenesis, dysregulation of *RB1*’s inhibitory actions on *E2F1*, a putative size ruler, are frequent oncogenic events (*27*). For example, many cancers have loss-of-function mutations in the *RB1* gene and/or exhibit up-regulated activity of extracellular signal–regulated kinase (*ERK*) kinases, which promotes increased *CCND1* levels and a concomitant increased activation of *RB1*’s inhibitor, *CDK4/6* (*28*). Mutations resulting in constitutively active B-Raf proto-oncogene, serine/threonine kinase (BRAF) or NRAS proto-oncogene, GTPase (NRAS) proteins, which result in increased ERK kinase activity and, ultimately, *CCND1* production (*29*), comprise 50% and 20% of all melanoma cases, respectively (*30*, *31*). These common driver mutations are likely to directly affect the size control machinery; however, the specific effect of these mutations on size control is essentially unknown.

Here, we leverage the natural phenotypic heterogeneity of a panel of melanoma cell lines to investigate the size scaling of intracellular peptides and transcripts in the context of cell growth and division. We show that *BRAF* and *NRAS* mutant melanomas have diverse mean sizes, but size uniformity is maintained. *RB1* subscales with size across lines. However, the relative ratio of *CCND1* and *RB1* is constant, suggesting that a common set point of *RB1* to *CCND1* is conserved, despite the presence of oncogenic mutations that can affect the levels of both proteins.

We identify sub- and super-scaling species across the cell proteome and phosphoproteome. In particular, we show that regulators of G_2_-M, translation, and growth subscale with size across lines, but proinflammatory proteins, extracellular matrix (ECM) components, regulators of the cytoskeleton, and certain growth factor receptors superscale. Through integration of transcriptomic data, we show that scaling of translation is regulated transcriptionally.

Larger lines, counterintuitively, have decreased levels of translation and altered biosynthetic signaling despite exhibiting an increased growth rate. Moreover, these lines continue to proliferate despite having sizes and phenotypes consistent with senescent cells. Mathematical modeling indicates that uncoupling growth and proliferative systems can facilitate division following a reduction mitogenic signaling. Conducting a regression analysis on the model parameters, we found that *TP53* or* CDKN1A/p21* expression covaries with promoters of growth. In proliferative large lines with senescent-like sizes and proteomes, either *TP53* or *CDKN1A/p21* is highly elevated. This research provides one of the first datasets describing how the transcriptional and proteomic profile of melanoma cells can change with cell size, indicating that cell morphology can have direct and meaningful effects on the chemistry of the cell.

## RESULTS

### Melanoma cell lines exhibit comparable size control but different cell sizes

To understand the relationship between cell size and different clinically relevant oncogenic drivers, we initially quantified the morphology of 17,547 single cells from 11 mouse melanoma cell lines from three different genetic backgrounds (data S1 and fig. S1). Lines were either: BRAF*, constitutively active* BRAF* typically due to a V600E mutation (*32*); NRAS*, constitutively active *NRAS* due to G12D mutations (*33*–*36*); or NRAS*/KDBRAF, where lines harbored a constitutively active *NRAS* mutation and a dominant negative mutation in the *BRAF* kinase domain (D594A) (subset of Table 1) (*34*). NRAS*/KDBRAF mutants mimic the clinical situation where there is paradoxical activation of *BRAF* following treatment of *NRAS* mutant cells with *BRAF* inhibitors such as vemurafenib (*37*).

**Table 1.** Cell line details.

Cell ID	Species	Genotype details	Derived from	Source
**19161**	**Murine**	**NRAS mutant (mut)/tyrosinase Cre recombinase A (CreA)**	**Brain melanoma**	**Pedersen *et al.* Cancer Discovery 2013** (*33*)
**19398**	**Murine**	**NRAS mut/tyrosinase CreA**	**Brain melanoma**	**Pedersen *et al.* Cancer Discovery 2013** (*33*)
**C873**	**Murine**	**NRAS mut/tyrosinase CreA**	**Brain melanoma**	**Pedersen *et al.* Cancer Discovery 2013** (*33*)
**Ear tum**	**Murine**	**NRAS mut/BRAF kinase dead (BRAF KD) under tyrosinase Cre recombinase-estrogen ligand-binding domain fusion protein (CreERT) with tamoxifen (TAM)**	**Cutaneous melanoma**	**Pedersen *et al.* PCMR 2014** (*34*)
**EAR B**	**Murine**	**NRAS mut/BRAF KD under tyrosinase CreERT with TAM**	**Cutaneous melanoma**	**Pedersen *et al.* PCMR 2014** (*34*)
**22532**	**Murine**	**NRAS mut/tyrosinase CreA**	**Brain melanoma**	**Pedersen *et al.* Cancer Discovery 2013** (*33*)
**14508 LN (A)**	**Murine**	**NRAS mut/BRAF KD under tyrosinase CreERT with TAM**	**Cutaneous melanoma**	**Pedersen *et al.* PCMR 2014** (*34*)
**17568**	**Murine**	**NRAS mut/tyrosinase CreA**	**Brain melanoma**	**Pedersen *et al.* Cancer Discovery 2013** (*33*)
**22783**	**Murine**	**NRAS mut/BRAF KD under tyrosinase CreERT with TAM**	**Cutaneous melanoma**	**Pedersen *et al.* PCMR 2014** (*34*)
**4434 (KB)**	**Murine**	**BRAF mut/p16** ^**−/−**^	**Cutaneous melanoma**	**Dhomen *et al.* Cancer Cell 2009** (*32*)
**17864**	**Murine**	**NRAS mut/UV**	**Cutaneous melanoma**	**Pedersen**
**17864A**	**Murine**	**NRAS mut/UV**	**Cutaneous melanoma**	**Pedersen**
**21917**	**Murine**	**NRAS mut/UV**	**Cutaneous melanoma**	**Pedersen**
**21015**	**Murine**	**BRAF mut/phosphate and tensin homolog (PTEN) null**	**Cutaneous melanoma**	**Pedersen**
**24038**	**Murine**	**NRAS mut/BRAF KD**	**Cutaneous melanoma**	**Pedersen *et al.* PCMR 2014** (*34*)
**B14341**	**Murine**	**NRAS mut/BRAF KD**	**Cutaneous melanoma**	**Pedersen *et al.* PCMR 2014** (*34*)
**C876**	**Murine**	**NRAS mut/tyrosinase CreA**	**Brain melanoma**	**Pedersen *et al.* Cancer Discovery 2013** (*33*)
**C790**	**Murine**	**NRAS mut/tyrosinase CreA**	**Brain melanoma**	**Pedersen *et al.* Cancer Discovery 2013** (*33*)
**17568**	**Murine**	**NRAS mut/tyrosinase CreA**	**Brain melanoma**	**Pedersen *et al.* Cancer Discovery 2013** (*33*)
**5555**	**Murine**	**BRAF mut/p16** ^**−/−**^	**Cutaneous melanoma**	**Dhomen *et al.* Cancer Cell 2009** (*32*)
**NRASQ61**	**Murine**	**NRAS Q61A**	**Cutaneous melanoma**	**Pedersen**

**Fig. 1.**
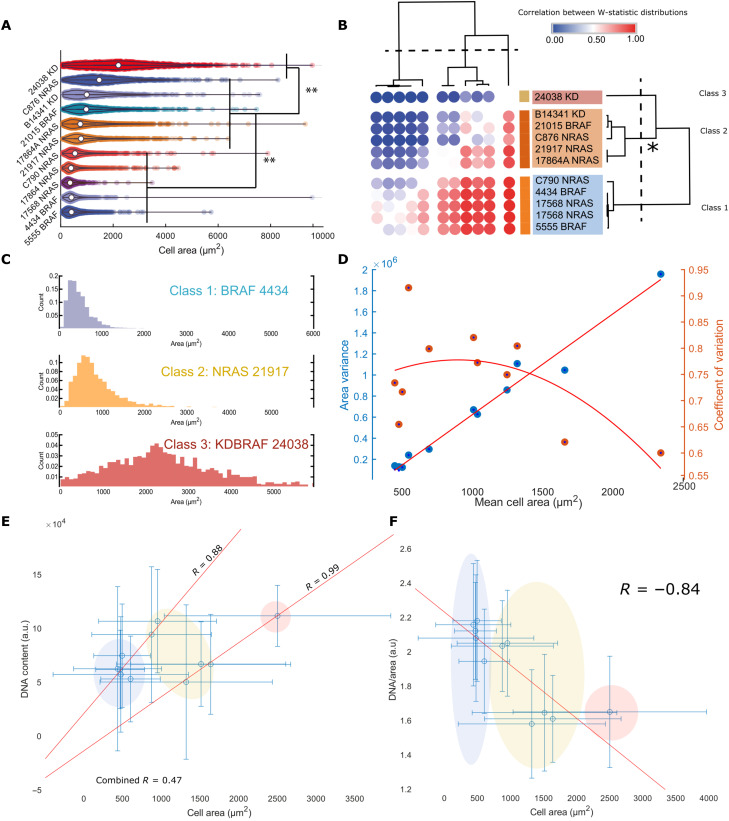
Melanoma cell lines exhibit comparable size control but different cell sizes; cell size relates to DNA content and DNA cytoplasm ratio. (**A**) Violin plot summarizing cell area distributions across lines. Acosh-normalized distribution means were subjected to an 11-way ANOVA test to confirm the significance of observed differences. ** indicates a *P* value < 0.01. (**B**) Heatmap showing a clustering of lines based on effects sizes calculated after Mann-Whitney tests (median uniqueness follows the same pattern as the means *P* < 0.01). Three distinct area classes emerge; the asterisk highlights that “class 2” seems the most variable. (**C**) Sample distributions from each class; from C1 to C3, skew decreases, while the means and variances increase. (**D**) Shows the relationship between the means and variances of the area distributions. The mean scales approximately linearly with variance (*R* = 0.93). The CV inconsistently varied with cell size. a.u., arbitrary units. (**E**) The relationship between mean cell area and mean DNA content, area weakly positively correlates with cell area (*R* = 0.47); however, two linear relationships appear to be present. Treating them separately drastically increases the correlation (*R* = 0.88 and 0.97). Error bars represent the SD of the single-cell data. Colored ellipses correspond to the “class” of the cell line. (**F**) Relationship between DNA per area and cell area. The two linear progressions in (E) are separated by DNA/cytoplasm ratio. Error bars represent the SD of the single-cell data. Colored ellipses correspond to the class of the cell line.

For each single cell, we quantified 60 features (*38*). We used the “cell area” feature as a proxy of cell size (*9*). Statistical analysis confirmed that the cell area distribution means were distinct [*N*-way analysis of variance (ANOVA), *P* < 0.05] (Fig. 1A), demonstrating extensive inter–cell line size heterogeneity. We performed an exhaustive series of Wilcoxon rank sum tests between area distributions (all distributions found to have unique medians, *P* < 0.001). By retrieving the *W* statistic, we calculated the “common language effect size” for each comparison. This produced a matrix of pairwise comparisons between all cell lines that measured the degree of difference in median area between them. Clustering the lines according to this difference, let us define three area classes (Fig. 1B): class 1: low mean (small cells), low variance, and high positive skew; class 2: moderate mean (larger cells), moderate variance, and moderate positive skew; and class 3: high mean (largest cells), high variance, and low skew (Fig. 1C). *BRAFKD*/*NRAS* cells tended to be larger. *NRAS* and *BRAF* active cells spanned the range of sizes. Although populations exhibited different extents of variance, we found that across all distributions, the mean cell area linearly scaled with the variance of cell area (*R* = 0.93), and the CV differed only modestly between cell lines (0.9 to 0.6, all cells; 0.4 to 0.6, G_2_) (Fig. 1D). This suggests that the lines have different size set points rather than altered control, as while their mean sizes are different, their relative dispersions about the mean remain approximately constant across the lines.

### Cell size relates to DNA content and DNA cytoplasm ratio in melanoma

Previous studies have indicated that DNA content (*3*) and concentration (*17*) are major determinants of cell size. Enacting fluorescence-activated cell sorting (FACS) analysis on a subset of our cell lines revealed that (fig. S2) both small [4434 (460 μm^2^) and 5555 (490 μm^2^)] and large [B14341 (1500 μm^2^) and 17864A (900 μm^2^)] cell lines are largely 2N, and all exhibit partial 4N populations. We note that 21917 (800 μm^2^) and 24038 (2400 μm^2^) were almost entirely tetraploid.

To further examine the relationship between DNA content and size in single cells, we then quantified the nuclear content (as judged by integrated Hoechst intensity) across lines. This metric differs from ploidy because it considers the amount of Hoechst staining within the nucleus, which can be affected by factors such as packing, but facilitates the measurement of many more cells. We observed two independent linear relationships between DNA content and cell area across cell lines (*R* = 0.47; *P* > 0.05, *n* = 11) when populations are pooled and (*R* = 0.88 and 0.99; *P* < 0.05, *n* = 7 and 4) when considered independently (Fig. 1E). Within cell lines, DNA content and size are linearly related, as expected (data S1). We then investigated how the DNA/cytoplasm ratio (D/C) scaled with cell size (*R* = −0.84, *P* < 0.05, *n* = 11) and identified two distinct clusters of cell lines: a set of smaller cell lines with a relatively high D/C and a set of large lines with a relatively low D/C ratio (Fig. 1F). The size “classes” identified prior correspond to a low DNA content, high D/C state (class 1); an overlapping high DNA content, low D/C or low DNA content, low D/C state (class 2); and a high DNA content, low D/C state (class 3).

Cell size in these cell lines is linearly related to DNA content. We hypothesize that this relationship can be shifted by changing the DNA cytoplasm ratio, implying two separate systems relating DNA and cell size in melanoma.

### Translation throttles CCND1 accumulation in response to upstream signaling

To understand the molecular drivers and consequences of size in our cell lines, we constructed a proteomic dataset capturing 9215 total peptides and identifying phosphorylation events on 4312 peptides, with a total of 21,355 unique phosphorylation events detected (data S2). Peptide expressions, normalized to reflect the relative difference in mass fractions across cells lines (Methods), were correlated to cell areas revealing proteins whose concentrations continuously scale with size.

**Fig. 2.**
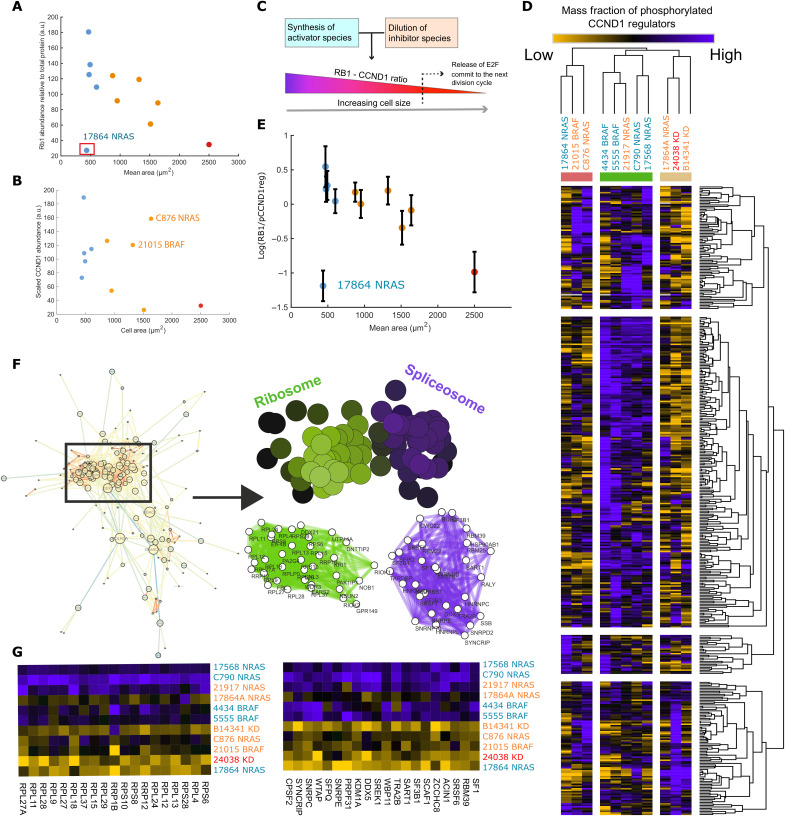
Translation throttles CCND1 accumulation in response to upstream signaling. (**A**) Negative correlation between *RB1* mass fraction and cell size (*R* = −0.84 (excluding 17,864, −0.51 if included), *P* < 0.05, *n* = 10 (or 11). (**B**) Relationship between *CCND1* mass fraction and cell size (*R* = −0.38, *P* > 0.05, *n* = 11), C876 and 21015 exhibit unexpectedly high levels of *CCND1* given their *RB1* abundance (**C**) Cartoon schematic depicting the role of *RB1* in the dilution model of G_1_-S transition and release of *E2F* transcription factor 1 (*E2F*). (**D**) Heatmap depicting the ratio of *RB1* against detected *CCND1* regulator phosphopeptides; ratios are typically lower in larger lines. (**E**) The average value for all RB1/rCCND1 ratios for each line plotted against cell area. Error bars represent ± 1 SD (*R* = −0.37 with 17,864, −0.86 without. *n* = 10 of 11, *P* > 0.05 (with), *P* < 0.05 without). (**F**) Network describing interactions between proteins correlating with *RB1*/p*CCND1*reg. SAFE overlay in (F) screening for graph regions enriched for ontological labels. Color intensity denotes the confidence of the enrichment (minimal enrichment confidence of 1 × 10^−3^). (**G**) Heatmaps showing the expression of peptides found in enriched regions of the interaction network across lines.

Previous studies have demonstrated that in normal cells, there is a critical *RB1 *concentration at which cells commit to division (*12*). We thought to investigate the expression of *RB1* and *CCND1* in our lines. Notably, 10 of 11 of the studied cell lines express detectable *RB1*, and mean *RB1* levels strongly subscaled with size [*R* = −0.84 (excluding 17,864, −0.51 if included), *P* < 0.05, *n* = 10 (or 11)] (Fig. 2A). Specifically, larger lines exhibited lower mean concentration of *RB1*. As within lines (*12*), *RB1* subscales with size between lines.

CCND1 abundance was found to be weakly negatively correlated with size (*R* = −0.38, *P* > 0.05, *n* = 11) (Fig. 2B). This suggests differential regulation of* CCND1* levels between lines, consistent with the presence of mutations across the mitogen-activated protein kinase (MAPK) pathway, rather than across sizes (see data S2 for phosphoproteomic data on the MAPK pathway in these cell lines). Despite their size and low *RB1* mass fraction, 21015 *BRAF* and C876 *NRAS* exhibited a *CCND1* mass fraction comparable to smaller cell lines, whereas our similarly large KD*BRAF* cell lines, 24038 and B14341, showed the lowest *CCND1* expression (ANOVA, KD*BRAF* versus *NRAS *+ *BRAF*, *P* < 0.05 *n* = 2, 9) (Fig. 2B). The ratio of *RB1 *to *CCND1* is largely uncorrelated with size (*R* = 0.2, *P* > 0.05, *n* = 11) (data S2). We concluded that the set point, where *CDK4/6:CCND1* activation exceeds *RB1* activity to putatively drive proliferation, is thus similar across lines.

To understand the molecular basis for the interline scaling of *CCND1*, we established a method to quantify the signaling activity upstream of *CCND1* using the phosphorylation state of transcriptional regulators of *CCND1*, as defined by the ENCODE database (*39*), (henceforth labeled* CCND1*regs) across different melanoma lines. All phosphorylations used in the analysis with known causative kinases or documented cellular effects [as determined via the PhosphoSitePlus (*40*) database] are detailed in data S4. These include several canonical upstream regulators of *CCND1* transcription such as *BRAF*–*MAPK* kinase–*ERK* as well as *JUN* and *MYC* among others. Across lines, we found that the phosphorylation of the majority of CCND1regs follows a similar trend to RB1 rather than CCND1 expression, negatively correlating with size (Fig. 2D; see data S2 for specific examples). These pathways were largely up-regulated in small cells (class I), consistent with the presence of activating mutations in BRAF and NRAS (Table 1). These pathways were also down-regulated in large cells (class 2/3), consistent with the fact that some of these lines have inactivating mutations in BRAF abolishing kinase activity (*34*, *37*).

We observed a negative relationship between cell size and the ratio between *RB1* and* CCND1*reg expressions, showing that larger cell lines, in fact, exhibit more pro-*CCND1* signaling per molecule of *RB1* than smaller cell lines [*R* = −0.37 with 17864; −0.86 without; *n* = 10 of 11, *P* > 0.05 (with); *P* < 0.05 (without)]. Moreover, this implies that the *CCND1* levels in larger lines are not due to differences in mitogen signaling alone.

Investigating this phenomenon, we identified proteins whose expression correlated with the *RB1*/p*CCND1*reg ratio (Fig. 2E) and conducted SAFE and ontological analysis [hit peptides satisfy; fold-change (Methods) of >1.5 or <0.66, |*R*| > 0.55] (Fig. 2F). We observed that low *RB1*/pCCND1reg ratio (i.e., in large cells) is associated with lower expression of ribosomal and spliceosome proteins (“translation” mRNA processing; e.g., *RPL26*, *SNRPE*, *P* < 10^−6^) (ontological enrichment significances are calculated using the hypergeometric test) (Fig. 2G and data S7). This suggests that reduced biosynthesis inhibits the conversion of *CCND1* signaling to functional *CCND1*.

Together, these data suggest that in large cells, while upstream activity of *CCND1* regulators is high relative to *RB1*, decreased translational efficiency can “throttle” *CCND1* protein accumulation. Subscaling of the biosynthetic fraction may exacerbate the effects of genetic factors limiting *CCND1* production, such as the kinase-dead *BRAF* mutation, D594A.

### Proteome-wide identification of sub and superscaling factors

We next sought to describe more broad differences in protein expression between the lines. We conducted a volcano analysis comparing correlations of protein mass fractions with size and the fold change across big and small cell lines (F3A).

**Fig. 3.**
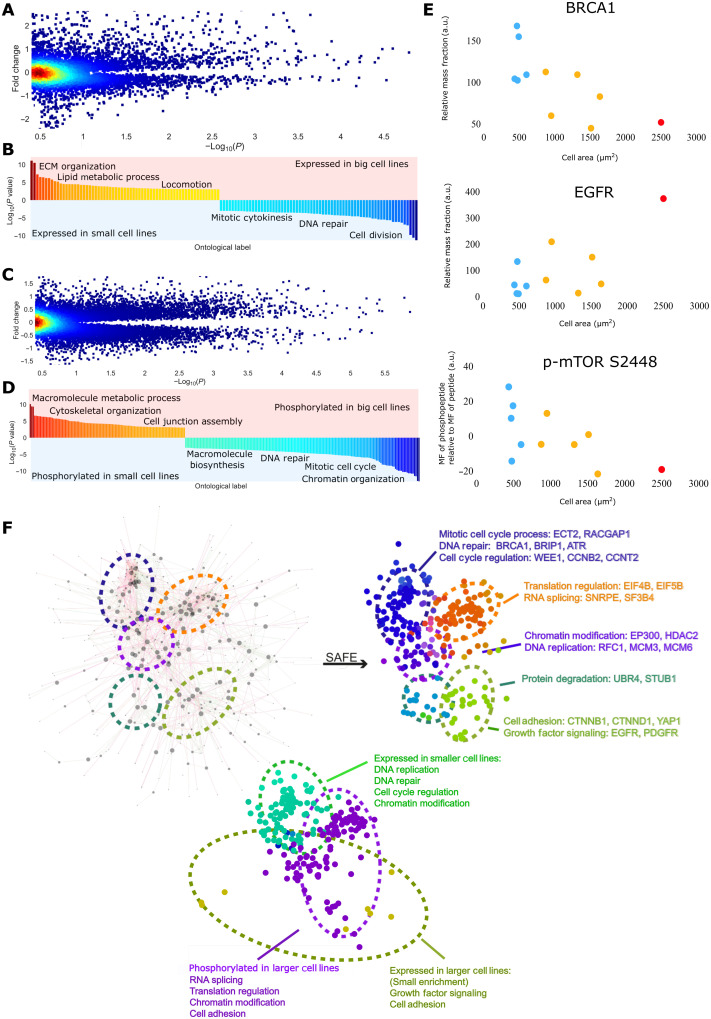
Proteome-wide identification of sub- and superscaling factors. (**A**) Fold change in peptide abundance across large and small cell lines plotted against the significance of the expressions correlation with size {genes achieving abs[log_2_(fc)] > 0.5, *P* < 0.05 are taken forward for ontological analysis}. Color represents data point density. (**B**) Ontologies enriched in peptides differentially expressed across big/small lines. (**C**) Fold change in phosphopeptide abundance across large and small cell lines plotted against the significance of the expressions correlation with size {genes achieving abs[log2(fc)] > 0.5, *P* < 0.05 are taken forward for ontological analysis}. Color represents data point density. (**D**) Ontologies enriched in phosphopeptides differentially expressed across big/small lines. (**E**) Example hits from the analysis,* BRCA1*, and relative *mTOR* phosphorylation subscale with size, *EGFR* superscales. (**F**) Network derived from screening for interactions within the list of size predicting, kinase-regulated, peptides. Interaction data were obtained from the STRING database. Right: SAFE overlay in (D) screening for graph regions enriched for ontological labels. Bottom: SAFE overlay in (D) screening for regions with high expression/phosphorylation in large/small cell lines.

We classified size-correlated peptides as “hits” (Fc > 1.5 or < 0.66, |*R*| >0.55), which were then sorted into two groups. One group of peptides are those expressed in class 1/2 (small) cells (thus, subscaling) and those expressed in class 2/3 (large) cells (superscaling). We conducted an ontological analysis on all the hit peptides (Fig. 3B). Small cells were enriched for subscaling proteins encoding regulators of cell cycle and mitotic processes (labels include “cell cycle process,” “mitotic cell cycle process,” and “cell division”; *P* <10^−9^). These proteins included checkpoint mediators *ATM*, *BRCA1*, and *WEE1* and the mitotic cyclin *CCNB2*. In contrast, class 2/3 large lines were statistically enriched for superscaling peptides from lipid/glycolipid metabolic processes and components of the ECM. These included *MVK*, *MVD*, *ACAT2*, and *COL2A1* (all ontology enrichments significant to at least *P* < 10^−3^) (data S3 and S4; see Fig. 3E for examples). To capture how protein kinase activity may also sub or superscale, we analyzed the set of proteins for which at least one phosphorylation was detected using the same system as that above (Fig. 3, C and D). In class I small cells, we observed a clear enrichment of subscaling phosphopeptides from cell cycle regulators (mitotic cell cycle, cell cycle process, and cell cycle; *P* <10^−9^, e.g., *BRCA1*, *SPDL1*, and *LIG1*) and biosynthetic processes [“regulation of cellular biosynthetic process,” “regulation of macromolecule biosynthetic process,” and “positive regulation of RNA metabolic process”; *P* <10^−9^, e.g., mechanistic target of rapamycin kinase (*mTOR*), *TSC1*, *EIF4B*, *EIF4G1*, and *MED26*], including the canonical mTORC1-activating phosphorylation, S2448(**49**) , implying up-regulation in small cell lines. Class 2/3 larger cell lines were enriched for superscaling phosphorylations on guanosine triphosphatase (GTPase) and cytoskeletal regulators (“positive regulation of GTPase activity,” “cell junction assembly,” and “regulation of cytoskeleton organization”; *P* <10^−4^, e.g., *CTNNB1*, *LATS1*, *ROCK1*, *ARHGEF5*, *ARFGEF1*, and *ARHGAP12*) and also set of growth regulators [“regulation of macromolecule biosynthetic process”; *P* <10^−6^, platelet derived growth factor receptor alpha, platelet derived growth factor receptor beta, insulin receptor substrate 1 (*IRS1*), protein kinase C delta, and DEP domain containing MTOR interacting protein (*DEPTOR*)], implying differential regulation of biosynthesis across the size range (data S3; see Fig. 3, E to G for examples). These included activating phosphorylations on *ROCK1* (S1341) and a *CTNNB1* degradation signal (S29) (*40*). Notably, *DEPTOR* phosphorylation and expression superscaled with size, implying down-regulation of insulin-TOR signaling; this is consistent with the observed reduction in *mTOR* S2448 expression. See figs. S4 and S5 for a full set of *mTOR *and cytoskeletal phosphorylations associated with cell size.

We then sought to define a regulatory network of proteins that scale with size. By integrating protein-protein interaction data (*41*) (see data S2 for these unfiltered hits) with our list of phosphopeptides and peptides whose mass fractions correlated with size, we derived a protein-protein network (*62*) (Fig. 3H). At this stage, we replaced scaled abundance of the phosphopeptide with an “adjusted abundance,” measuring the phosphopeptide abundance relative to the amount of peptide detected (Methods). This way, we could reveal peptides that were more/less phosphorylated than expected, given their mass fraction.

Application of the SAFE algorithm (*42*) visualized themes embedded in the networks of super- and subscaling proteins (Fig. 3H) (*43*–*46*). Analysis of these networks, in conjunction with an additional ontological analysis, echoed the prior results, that it is the disproportionate expression and phosphorylation of “G_2_-M control” phosphopeptides that defines our smaller cell lines (e.g., *BRCA1*, *WEE1*, *CCNB2*, and *ATM*), including *BRCA1* S686 an *AKT1* target and stabilizing phosphorylation (*40*). In larger lines, we observed altered phosphorylation of “translation control” (e.g., *eEIF4B* and *EIF5B*), “spliceosome machinery” (e.g., *SNRPE* and *SF3B4*), “cell adhesion” peptides (e.g., *YAP1*, *YES1*, and *CTNND1*), and increased expression of “growth signaling” (e.g., epidermal growth factor receptor and platelet-derived growth factor receptor) peptides (Fig. 3H) (all enrichments significant to least *P* < 10^−3^). Despite *mTOR* S2448 subscaling, many phosphorylations enriched in larger cell lines correspond to insulin signaling events such as *EIF4B* S497 (*40*). The consequences of many such phosphorylations are unknown, but given the subscaling of *mTOR* S2448, they likely fulfill inhibitory functions.

Larger cell sizes, related to lower D/C ratios, are associated with decreased G_2_-M and DNA-Damage response (DDR) mass fractions and increased relative expression of ECM and lipid metabolic components. Further, large sizes coincide with increased phosphorylation of cytoskeletal effectors and differential regulation of the biosynthetic machinery.

### Inflammatory transcripts are enriched in larger cell lines

**Fig. 4.**
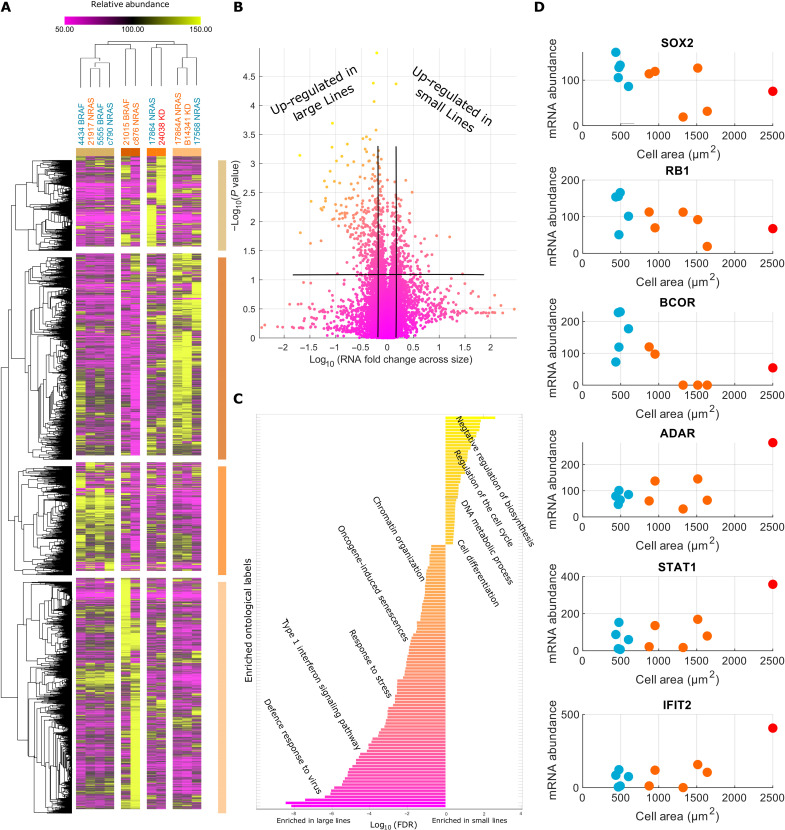
Inflammatory transcripts enrich in larger cell lines. (**A**) Heatmap of transcriptomes across cell lines. Lines/transcripts are grouped through hierarchical clustering conducted using the “Morpheus” software (Broad Institute). (**B**) Volcano plot for the fold change of transcripts across size groups against the RNA size correlation. The genes achieving abs[log2(Fc) > 0.5] and |*R*| > 0.55 were taken forward for ontological analysis. (**C**) Ontologies enriched in large/small cell lines. Inflammatory transcripts enrich in larger lines, while those related to cell cycle and gene regulation enrich in smaller lines (FDR < 1 × 10^−3^). (**D**) Examples of correlating transcripts in either group.

We next performed transcriptomic experiments to gain further insight into the relationship between size and signaling network organization and activity. We measured the abundance of 24988 RNA molecules overlapping with 9290 measured peptides (data S5). We conducted a volcano analysis, as described previously (Methods), to identify a list of transcripts that super- and subscale with size across lines (Fig. 4A). mRNA’s relating to cell cycle regulation (“regulation of cell cycle”;* BARD1, WEE1, E2F8, *and* RB1*) and control of gene expression (“negative regulation of gene expression,” “chromatin organization”, and “cell differentiation”; e.g., *SIN3A, SOX2, *and* HMGA1*) subscaled with cell size (enrichments significant to at least *P* < 1 × 10^−3^) (Fig. 4, B and C, and data S3). These observations are in line with observations that small cells express relatively higher levels of cell cycle regulatory proteins such as *WEE1*. Transcripts pertaining to inflammation processes [“inflammatory signaling” and “interferon signaling” including signal transducer and activator of transcription 1 (*STAT1*), interferon induced protein with tetratricopeptide repeats 1, interferon regulatory factor 5 (*IRF5*) and *IRF7*, and adenosine deaminase RNA specific] were up-regulated in larger cell lines (enrichments significant to at least *P* < 1 × 10^−3^).

In conjunction with that observed in the proteomic data, these data show that compared to class 1/2 smaller cells, class 2/3 larger cells have decreased levels G_2_-M regulators, altered metabolism and biosynthesis, and an increased inflammatory (transcript) mass fraction.

### Transcription regulates ribosomal scaling

**Fig. 5.**
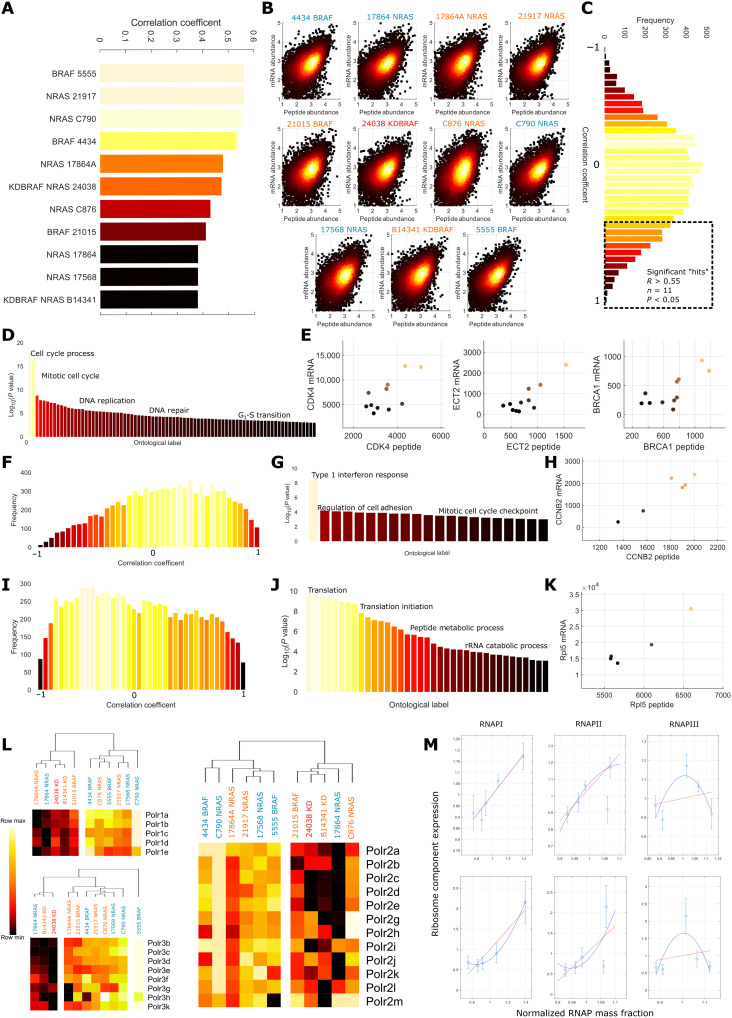
Transcription regulates ribosomal scaling. (**A**) Correlation between gene and peptide expression within each cell line; coefficients range between 0.4 and 0.6. (**B**) Log-log plots of RNA against peptide abundance; color intensity is proportional to the density of the data points. (**C**) Distribution of correlation coefficients between peptide and mRNA abundances across cell lines, dotted box indicates genes with significant (*P* < 0.05, |R| > 0.55, *n* = 11) positive correlations. (**D**) Enriched gene ontologies detected in the genes with significant RNA-peptide correlations (*P* < 1 × 10^−3^ enrichment confidence or higher). (**E**) Example protein-mRNA correlations from the cell cycle process and mitotic cell cycle process themes. (**F**) Distribution of correlation coefficients between mRNA and peptide abundances in small (area < 900 μm^2^) cell lines. We note a positive skew and more positive mean than in the pooled distribution. (**G**) Themes enriched in the set of genes exhibiting significant correlations (*P* < 0.05, *n* = 6, |*R*| > 0.70) between peptide and mRNA abundance in small cell lines. (**H**) Example correlation between* CCNB2* peptide and mRNA abundance from the mitotic cell cycle checkpoint theme. (**I**) Distribution of correlation coefficients between mRNA and peptide abundances in big (area > 900 μm^2^) cell lines. We note a negative skew and more negative mean than in the pooled distribution. (**J**) Themes enriched in the set of genes exhibiting significant correlations (*P* < 0.05, *n* = 5, |*R*| > 0.75) between peptide and mRNA abundance in big cell lines. (**K**) Example correlation between *Rpl5* peptide and mRNA abundance from the translation theme. (**L**) Expression of all detected RNA Pol 1 (top left)/2 (right)/3 (bottom left) components across cell lines. Large lines tend to exhibit lower expression. (**M**) Relationship between RNA Pol 1/2/3 (left to right) peptide expression and the peptide (top)/mRNA (bottom) expression of identified RNA peptide correlates in large cells in the translation theme. We note that both RNA and peptide abundance correlate, suggesting transcription regulation of peptide expression.

To examine the role of translation and transcription in size and proliferation control, we first related mRNA and peptide abundances in each line. Correlation coefficients between mRNA and expression ranged between 0.56 and 0.38 (*n* = 9290), in agreement with previous studies (*47*) (Fig. 5, A and B). We then calculated correlations at the gene level, across cell lines. Notably, this revealed that for most genes, there is poor correlation between mRNA fraction (reads of gene/total reads in the cell line) and peptide mass fraction. Of those that exhibited significant correlations (1116 of 9290), many showed negative relationships (277 of 1116) (Fig. 5C).

By conducting ontological analysis on the genes with significant, (*P* < 0.05, |*R*| > 0.55, *n* = 11) correlations with peptide abundances, we observed an obvious enrichment of cell cycle and DNA repair/replication genes (cell cycle process, “DNA replication,” and “mitotic cell cycle phase transition”; *P* < 10^−6^, e.g., *“BRCA1,” “CCNB2,” “CCND2,” “CCNA2,” “BRIP1,” “CDK4,” *and* “ECT2”*) (Fig. 5, D and E). As peptides are synthesized from transcripts, a correlation between peptide and mRNA mass fractions implies that transcription is limiting the production of these proteins. We note that uncorrelated peptide-mRNA abundances do not necessarily indicate translation limiting peptide manufacture, as mRNA-peptide correlation can be “buffered” through several other means, such as protein degradation (*48*).

To identify genes that were strongly correlated in specific size classes, we split the transcript/proteomic datasets into “large” and “small” subsets, composed of cell lines with sizes above/below the mean (900 μm^2^), and recalculated the correlation coefficients between mRNA and peptide fractions. At the gene level, in the smaller subset, we observed enrichment of inflammatory, adhesion and cell cycle regulators (“type 1 interferon signaling pathway,” “regulation of cell adhesion,” and “mitotic cell cycle checkpoint”; *P* <10^−5^, e.g., *“IRF9,” “IFIT3,” “STAT1,” “RHOD,” “CTHRC1,” “CCNB2,” *and* BRCA1*) (Fig. 5, F to H) in the list of genes with significant peptide-mRNA correlations (*n* = 5, *P* < 0.05, |*R*| > 0.75). In large cells, there was a strong correlation between mRNA and the protein mass fraction of ribosomal and translational genes (*n* = 5, *P* < 0.05, |*R*| > 0.75) (translation, “cytoplasmic translation,” and “cytoplasmic large ribosomal subunit”; *P* <10^−9^, e.g., *RPL26, RPL8, RPL23, RPL5, *and* ETF1*) (Fig. 5, I to K). This suggests that control of the expression of translational components occurs through transcription at larger cell sizes. Correlating RNA-polymerase (RNAP) 1/2/3 component expressions (Fig. 5L) to the mRNA abundance of ribosomal components, we note a clear positive relationship (for Pol1/2). This extended to the peptide abundance, further suggesting that the production of ribosomal peptides is transcriptionally limited in these cell lines. This was not observed in smaller cell lines; while the correlation between Pol1/2 expression and peptide expression was maintained, the relationship with mRNA was disrupted, indicating a translational, or at least, posttranscriptional dependency in smaller lines (data S5 and S3).

Together, these data suggest that the disruption of cell transcription relates to the subscaling of the biosynthetic mass fraction in larger, low D/C, cell lines. This may occur through the decreased RNAP expression at larger sizes. Depressed biosynthetic expression and signaling may underpin further scaling relationships.

### Validation of size-scaling relationships in an independent panel of melanoma cell lines

To assess the universality of our size-scaling relationships, we extended our panel of 11 lines to include a further 12 composed of the same genotypes as before and conducted further quantitative morphological and phosphoproteomic experiments and analyses (statistical thresholds for hit detection remain the same as the previous analysis). In contrast to the previous analysis, we note an additional “arm” of the volcano plot indicating a subset of peptides extremely enriched in larger cell lines (fig. S3). We hypothesize that this represents gene overexpression rather than superscaling relationships, as there is no smooth transition in between the arms. Initially including these genes in the analysis, we found that an increased mass fraction of apoptotic effectors [“apoptotic signaling pathway,” “positive regulation of mitochondrial membrane permeability involved in apoptotic process,” and “necrotic cell death”; *P* < 10^−3^, e.g., “*BOK*,” “Toll-like receptor 3 (*TLR3*),” “*TLR4,” “BCL2,*” and *“TICAM1*”] and lipid/carbohydrate metabolism (“lipid metabolic process,” “small-molecule metabolic process,” and “oligosaccharide metabolic process”; *P* <10^−8^, e.g., *GAA, NEU1, ALG11, *and* ACOX3*) is associated with large cell lines. Excluding the “overexpressed” genes, we observe enrichment of lipid metabolic proteins alone (“lipid biosynthetic process,” lipid metabolic process, and “sterol metabolic process”; *P* < 10^−5^). In contrast, we observe a clear enrichment of cell cycle (cell cycle process and cell cycle checkpoint; *P* <10^−15^, e.g.,* CDK2, CCNB2, CCNA2, *and* CDC45*), mitotic (mitotic cell cycle checkpoint and “chromosome segregation”; *P* < 10^−10^, e.g., *SPDL1, ECT2, *and* PLK1*) and DNA repair (“DNA repair” and “cellular response to DNA damage”; *P* < 10^−10^, e.g., *BRCA1 *and* LIG1*) peptides in smaller lines indicating subscaling (fig. S4 and data S3).

Enacting the same analysis for the phosphopeptides (fig. S4), we again observe additional arms, indicating gene overexpression in big/small cell lines. We first calculated enrichments for the two central arms finding phosphorylations on cell cycle, DNA repair, and biosynthetic regulatory peptides (cell cycle process, DNA repair, and regulation of macromolecule biosynthetic process; *P* < 10^−7^, e.g., *RCA1, CHEK1, CDK4, EIF4B, “RPS5,” *and *EIF3G*) enrich in smaller cell lines. In larger cell lines, phosphorylations pertaining to cytoskeletal and growth factor signaling (regulation of GTPase activity, “cytoskeletal organization,” cell adhesion, and “regulation of epidermal growth factor receptor signaling pathway”; *P* < 10^−3^, e.g.,* ARHGEF6, GIT1, TSC2, ROCK1/2, CDC42, TLN1, *and* AKT1/3*) are enriched. Within each overexpressed group, we found that larger cell lines were up-regulating GTPase signaling elements (positive regulation of GTPase activity, “regulation of small GTPase–mediated signaling,” and “Rho protein signal transduction”; *P* < 10^−10^, 10^−10^, 10^−10^, respectively), e.g., *ARFGAP1, TIAM2, *and* ARHGAP1*, while up-regulations in smaller cell lines followed no theme (fig. S3 and data S3). We then investigated which ontological themes were enriched in both analyses finding good agreement; a full discussion of this analysis can be found in the Supplementary Materials. We recover a large, *BRCA1*-centric set of interacting genes in both analyses, implicating *BRCA1* in size-dependent phenomena (fig. S3).

These data corroborate our previous analysis, strengthening the claim that G_2_-M and DNA repair processes define smaller melanoma cell lines (with associated peptides subscaling with cell size), while regulation of cytoskeletal organization and the rewiring of biosynthetic signaling and lipid metabolism define larger cell lines (peptides superscaling with size).

### Cell growth rate scales with cell size despite down-regulation of biosynthetic effectors

**Fig. 6.**
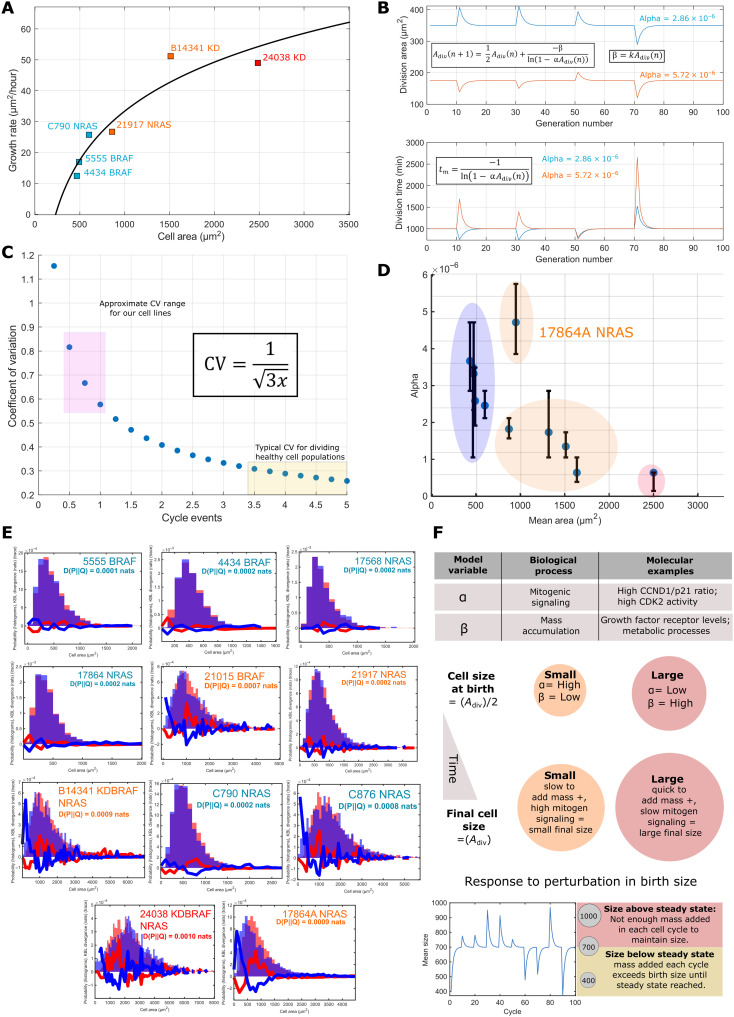
Theoretical modeling suggests decoupling of low mitogen and growth signaling drive proliferation of larger cells. (**A**) The relationship between cell growth rate and cell size across six lines representing each genotype and size class. Growth rate increases with increasing cell size. (**B**) A demonstration of the stability of the size distribution mean to perturbations to a cell division area and instability of the mean with respect to perturbations to α. Bottom depicts the stability of proliferation rate with respect to both parameters. (**C**) The function describing how the CV changes with increasing cycle complexity; the pink box marks the CV’s observed in our cell lines and the yellow, those observed in other studies. (**D**) The relationship between fitted α values and cell size, α broadly negatively correlates with the mean size of the cell line (*R* = 0.75, *n* = 11, *P* < 0.05). Error bars indicate the range of α values that generated similar fit strengths. (**E**) Model outputs demonstrating the best fits achieved for G_1_ distributions. Orange histograms are model outputs, and blue histograms are the experimental data. The blue line shows D(experimental||measured) and the red line the reverse. (**F**) Cartoon summary on the model relating parameters to biologically processes.

Having observed subscaling of ribosomal and spliceosome peptide expression, differential phosphorylation of biosynthetic regulators, depressed proliferative signaling, and an enrichment of inflammatory effectors in larger cell lines, we expected them to exhibit a notably decreased growth rate. To investigate this, we live-imaged two cell lines from each genotype spread across the observed range of cell sizes and quantified the average rate of growth as the area gain per time, (square micrometer per hour) (Methods). Unexpectedly, growth rate was found to increase with cell size despite the observed down-regulation of biosynthetic effectors (Fig. 6A), and proliferation rate was only modestly affected (fig. S3). We note, however, that this relationship does not appear linear, suggesting that the system behind this phenomenon begins to fail at large cell sizes. These data show that larger melanoma cell lines can maintain cell growth without the scaling of classical growth regulators.

Investigating *mTOR* signaling specifically, we note that while the primary activating *mTOR* phosphorylation sites [S2448, S2481; phosphorylations responsible for signaling through mTORC1/2, respectively (*49*)] are under-phosphorylated in larger cells (phosphopeptide abundance is lower than expected given peptide abundance), many upstream regulators exhibit phosphorylations typical of insulin-driven receptor tyrosine kinase (RTK) signaling. However, these genes were differentially phosphorylated across the cell sizes; for example, *IRS1* S414 is enriched in smaller cell lines, while *IRS1* T448 is enriched in larger lines (*40*). These data further indicate that differential, rather than reduced, RTK signaling across sizes leads to the observed down-regulation of biosynthetic effectors in larger cell lines (fig. S5).

Given the altered signaling state and having noted an increased cytoskeletal peptide mass fraction (and an increase in its phosphorylation) in larger cell lines, we were interested in the state of canonical RTK-driven pathways of cytoskeletal activation. We noted that *HER2*, (1108),* SRC* (S17), *PAK4* (S476), *ROCK1* (S1341), *VASP* (S317), and *LIMK1* (S298) [many of which are activating phosphorylations (*40*)] among others, where disproportionately abundant in larger cell lines, indicating that RTK-driven cytoskeletal activity is up-regulated (fig. S6). We hypothesize that larger cell lines have skewed their RTK signaling machinery toward the regulation of cytoskeleton reorganization rather than through up-regulation of anabolic processes. This may lead to increased size, in part, by increased spreading of the cell.

### Theoretical modeling suggests decoupling of low mitogen and growth signaling drive proliferation of larger cells

Noticing that growth rate is maintained in larger cell lines in the background of down-regulated proliferative and biosynthetic signaling, we sought to understand the significance of this effect at a more system level. We used a simplification of recent models, where the transition rate between cell cycle stages is governed by a power-law relationship with cell size

$$R=\alpha A{\left(t\right)}^{y}$$ (1)

Following previous studies (*50*), the transition time probability distribution under an exponential growth condition is given as

$$A(t)=A_{b}e^{kt},P(T>t)=e^{-\int_{0}^{t}\alpha A{\left(s\right)}^{y}ds}=e^{-\frac{\alpha V_{b}^{\gamma }}{k\gamma }(e^{\alpha \gamma t}-1)}$$ (2)

indicating that the γ^th^ power of the added area follows an exponential distribution centered on γ*k*/α;

$$P[V_{b}^{\gamma }(e^{\alpha \gamma t}-1)>t]=e^{-\frac{\alpha t}{k\gamma }}$$ (3)

Taking *y* = 1, the mean added mass equals *k*/α. We could capture similar behavior when considering the simpler case where the probability of transitioning between cycle stages and growth rate are taken to be a constant within a cycle but are adjusted according to cell division size (*A*_div_), (*P* = α*A*_div_, β = *kA*_div_, respectively) defining a Poissionian system. We believe that this simplification provides a useful tool for the understanding of cell size determination in the adder case. Here, we have assumed adder-like behavior, as small errors in sizer mechanisms can lead to phenomenological adder systems (*9*). Using these, we could derive expressions for the expected proliferation rate and added size, given as exponential distributions

$$P(t)=\lambda e^{-\lambda t},\lambda =-\frac{1}{\ln(2)\;}\ln\;(1-\alpha A_{\mathrm{div}})$$ (4)

$$P[A(t)]=\lambda_{2}e^{-\lambda_{2}A(t)},\lambda_{2}=-\frac{1}{\ln(2)\;A_{\mathrm{div}}k}\ln(1-\alpha A_{\mathrm{div}})$$ (5)

We include the derivations in the Supplementary Materials. This facilitated the construction of a simple system of equations dictating cell size

$$S(\alpha ,A_{\mathrm{div}},k)=\{t_{m}=\frac{-\ln(2)}{\ln[1-\alpha A_{\mathrm{div}}(n)]},\;A_{\mathrm{div}}(n+1)=\frac{1}{2}A_{\mathrm{div}}(n)+\frac{-\ln(2)\beta }{\ln[1-\alpha A_{\mathrm{div}}(n)]},\;\beta =kA_{\mathrm{div}}(n)\}$$ (6)

where “*t*_m_” is the mean proliferation time, and “*n*” is the number of proliferative cycles that have passed. Perturbing the parameters of this system, we find that it is stable to perturbations in *A*_div_ (Fig. 6B) but unstable to changes in α or *k*. That is, a constant mean size is maintained under this system that may be adaptively regulated by modification of α, related to mitogenic signaling and *k*, controlling the growth rate.

If α is perturbed, then the proliferation rate initially decreases but exponentially decays back to the initial value across successive division cycles. Thus, if the α and *k* parameters are independent, then cell growth provides a means to “correct” proliferation rate under perturbation to mitogenic signaling (Fig. 6B).

Using Eqs. 4 and 5, we could derive the moments of the expected size distribution (see the Supplementary Materials). This is a hypo-exponential function, with a mean and variance given as

$$\langle P[A(t)]\rangle =\frac{2x}{\lambda_{2}}=\frac{2x\;k\ln2}{\alpha },\langle \langle P[A(t)]\rangle \rangle =\frac{4x}{3{\left(\lambda_{2}\right)}^{2}}=\frac{4xk^{2}{\left[\ln(2)\right]}^{2}}{3\alpha^{2}}$$ (7)

where *x* is the number of “stages” in the model of cycle. This yields a CV

$$\mathrm{CV}=\frac{\sqrt{\frac{4x}{3{\left(\lambda \right)}^{2}}}}{\frac{2x}{\lambda }}=\frac{1}{\sqrt{3x}}$$ (8)

The cell lines have G_1_ CVs of ~0.7 to 0.5, and *x* was calculated to range between 0.8 and 1.1. For simplicity, we took *x* = 1 from this to avoid complications stemming from a decimal number of cell cycle stages. We note that this approach is only feasible when the CV > ~0.25 as the differences in CV values for neighboring “*x*” tend to become 0 as *x* increases; this is equivalent to an ~5-stage system (Fig. 6C).

These results allow us to define a simple and efficient algorithm to calculate predicted cell size distributions (Methods) (Fig. 6E). The α values were fit to experimentally determined area distributions by minimizing the Kullbeck-Leibler divergence between measured and calculated distributions (β is a measured parameter for 6 of 11 cell lines; the remaining lines growth rates are fit to the measured relationship); α values exponentially decrease with increasing cell size (Fig. 6F and Table 2). The simulation accurately recapitulated much of the measured data (total G_1_ and G_2_ distributions); however, in the case of larger cell lines, the model partially underpredicted the abundance of small (A < 500 μm^2^) cells [see 24038 (2500 μm^2^), C876 (1600 μm^2^), 17864A (1000 μm^2^), and B14341 (1500 μm^2^) (Fig. 6G)]. A summary of the model can be found in Fig. 6H. Together, our modeling has shown that given a proportionality between cell size and division probability, increased cell growth is an effective means of triggering proliferation when scaling of proliferative factors is perturbed, for example, by a reduction in mitogenic signaling.

**Table 2.** Model parameters.

Cell line	α (*P*/μm^2^)	β (μm^2^/min)
**4434**	3.49 × 10^−06^	0.16
**17864**	3.67 × 10^−06^	0.22
**17864A**	4.71 × 10^−06^	0.43
**21917**	1.82 × 10^−06^	0.35
**21015**	1.73 × 10^−06^	0.55
**24038**	6.38 × 10^−07^	0.49
**B14341**	1.35 × 10^−06^	0.52
**C876**	6.38 × 10^−07^	0.60
**C790**	2.46 × 10^−06^	0.25
**17568**	3.32 × 10^−06^	0.19
**5555**	2.59 × 10^−06^	0.20

### Expression levels of the TP53-CDKN1A-CCND1 axis predict cell growth rate and model parameter values

**Fig. 7.**
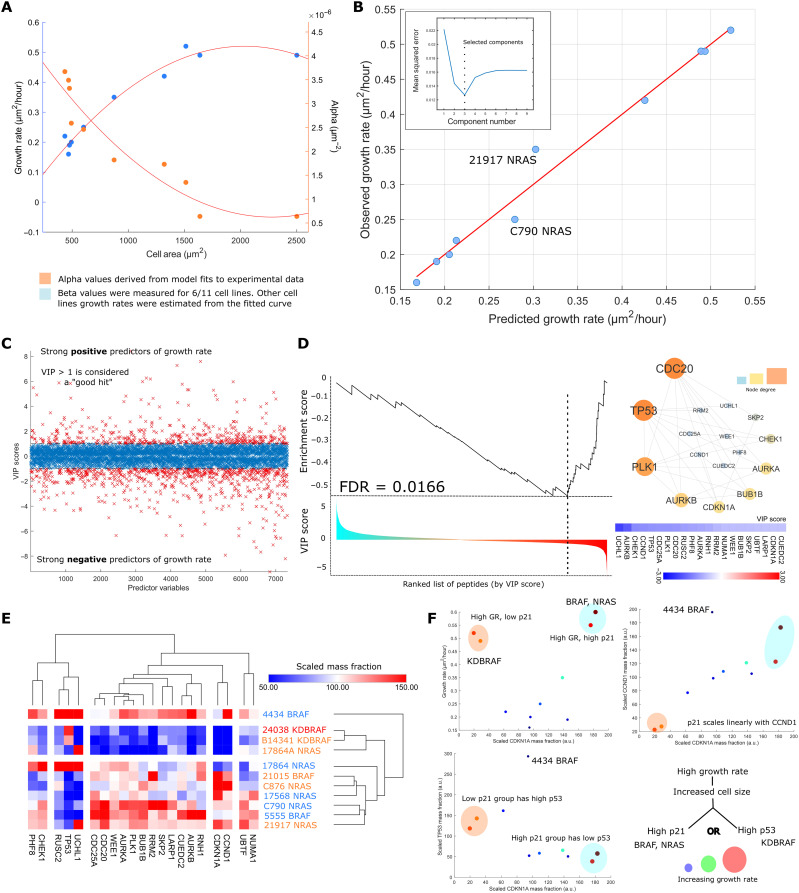
Expression levels of the TP53-CDKN1A-CCND1 axis predict cell growth rate and model parameter values. (**A**) Relationship between cell size and the α (mitogenic signaling) and β (growth rate) model parameters: Neither exhibits a linear relationship. Growth rates were interpolated from the size-growth curve for cell lines where a growth rate was not directly measured. (**B**) PLSR model predicting cell growth rate from proteomic expression data. The inset shows the relationship between mean squared error and component number through fivefold cross validation. Three components were chosen for this model corresponding to the minima of the curve. (**C**) VIP scores calculated for peptide with reference to the model in (B). The sign of the VIP score was artificially made negative if the weight of the peptide in the model first component was <0. (**D**) The enrichment score of the *p53-cdkn1a-ccnd1* signaling module as a function of the ranked position of all measured peptides. Node size and color in the accompanying network diagram are proportional to node degree. (**E**) The expression of the network components shown in (D) across lines. Note that not all members correlate with size but rather covary with size correlates. (**F**) Top left: Relationship between *p21* mass fraction and cell growth rate. Lines with the highest growth rates are circled for easier tracking through the subsequent graphs. Top right: Linear relationship between the *p21* and *CCND1* mass fractions. Bottom left: The* p21* mass fraction plotted against the *p53* mass fraction; *p53* and *p21* expression appear mutually exclusive in large cell lines. In all cases, size and color of the data points are proportional to cell growth rate.

We noted that the α (mitogenic signaling) and β (growth rate) parameters of our model showed nonlinear relationships with cell size (exponential and hyperbolic respectively); thus, we expected that peptides whose levels correlated with α and β would be (partly) independent of those that correlate with size as determined by imaging (Fig. 7A). Thus, by leveraging our proteomic datasets, we could provide mechanistic insight the coordination of size and growth.

We constructed a partial least-squares regression (PLSR) model predicting parameter values from the proteomic expression data (*R* = 0.98). Fivefold cross validation was used to select the number of components and maximize transferability of the model (Fig. 7B). For each peptide, we calculated a variable importance to projection (VIP) score (Methods), representing the significance of each peptide to the prediction of α and β; the sign of the VIP score was set to reflect the model weights of the first component (Fig. 7C). Gene set enrichment analysis (GSEA) (*51*) was used to find overrepresented proteins and complexes in lists of peptides predictive of α and β.

We found that down-regulation of an interaction module containing many canonical regulators of cell cycle progression were strong predictors of growth rate in our cell lines (similarly, an up-regulation–predicted α or extent of mitogen signaling) [false discovery rate (FDR) < 0.05], including *WEE1, SKP2, *and* CDC25A* (Fig. 7D). Expression of peptides pertaining to lipid metabolic processes and ECM deposition where positively predictive of growth rate (FDR, 0.05) (fig. S5). Proteins that covaried with these peptides included *CCND1, p21, *and* TP53* (Fig. 7D). Many of these peptides were also found to correlate with cell size (as determined by imaging). Thus, using an unsupervised, independent analysis, we showed that proteins that sub/superscale with size are also key regulators of growth. This suggests that in many cases, the concentration of many of these proteins, which is a function of size, will directly affect growth rates.

We hypothesized that proteins that predicted growth, or whose levels covaried with predictors of growth, could provide insight into mechanisms by which proliferation is maintained in cancer cells with relatively large cell areas and high cytoplasmic/nuclear ratios. In normal cells, large size can lead to senescence (*14*, *17*–*18*). We focused particularly on *TP53* and *CDKN1A/p21*, which covaried with proteins that are predictive of growth (Fig. 7, D and E). We observed a trimodal relationship between growth rate, *CDKN1A*, and *TP53* expression. Most cells had modest levels of *CDKN1A* or *p53* and average growth rates. However, two groups of cells had relatively high growth rates, corresponding to their increased size and high cytoplasmic/nuclear ratio (lines 21015 BRAF, C876 NRAS, B14341 KDBRAF, and 24038 KDBRAF). However, these lines were notably different in their levels of* CDNK1A* and *TP53*. One group of *BRAF*KD cells has high growth rates and large size and is *CDKN1A* low and *TP53* high. However, the other group of NRAS and BRAF mutant cells has high growth rates and large size and is *CDKN1A* high and *TP53* low. Further, *CDKN1A* and *CCND1* mass fractions were observed to scale linearly with one another (excluding 4434, *R* = 0.92, else *R* = 0.72, *P* < 0.05, *n* = 11). Thus, in these cell lines, a high growth rate (and large size) is associated with two distinct states: a /*TP53*low/*p21*high/*CCND1*high state and a /*TP53*high, /*p21*low/*CCND1*low state (Fig. 7F). Together, these data suggest that large melanoma cells exist in two states that increase the probability of cell cycle progression, and not senescence, despite subscaling behavior of the biosynthetic and cell cycle mass fractions.

## DISCUSSION

We have identified scaling relationships between cell size and peptide/gene expression in melanoma. Expression and phosphorylation of G_2_-M, DNA-associated and biosynthetic peptides exhibited a clear subscaling relationship with cell size across two independent panels of melanoma cell lines, while expression of lipid metabolic genes and phosphorylation of cytoskeletal regulators showed the reverse. This is in strong agreement with numerous recent studies investigating the relationships between cell size and gene/peptide expression, identifying histones as subscaling components (*15*), observing an up-regulation of lipid metabolism in larger cell lines (*17*), noting a decreased abundance of translational components and translation rate in large polyploid cells (*16*), and full proteome surveys of scaling components in lung fibroblasts (*14*) and a large panel of human cell lines (*18*).

We observed that the mean* RB1* mass fraction decreased with increasing cell size, corroborating the findings of recent studies associating *RB1* (and *Whi5*) dilution to size determination and control (*7*, *12*). This trend extended to the abundance of phosphopeptides associated with *CCND1* transcription and the abundance of core ribosomal and spliceosomal peptides. These data suggest that the state of the *RB1-CCND1* axis in melanoma or, indeed, an *RB1*-dilution system (*12*) is sensitive to both the strength of proliferative signaling and translational capacity of the cell in melanoma. Reduced signaling and protein production may decrease *CCND1* abundance, and therefore, *RB1* must dilute further to induce division commitment, thereby delaying proliferation until a greater cell size. Recent literature suggests G_2_-driven synthesis of *CCND1* (*13*, *51*), noticing a tight dependence on cellular translation (*13*). Translation and mitogen signaling in the prior G_2_ may color events in the subsequent G_1_. “Subscaling” of G_2_-M regulators, such as *WEE1* and *BRCA1*, may relate to smaller cells exhibiting increased expression of *CCND1*.

We constructed a simple theoretical model to demonstrate how continued growth under proliferative stress could maintain the cell proliferation rate. This relied on the probability of a cell transitioning to the next stage of the cell cycle being proportional to its size (*50*), for example, via *RB1* dilution. This is consistent with the recent observation that cell cycle phase lengths across generations are coupled in cancer cell lines (*52*), here, via mother cell size (*13*). The same study notes that this effect may be unique to cancerous cell lines due to a disproportional abundance of regulators acting at multiple stages of the cell cycle (*52*), in effect, “simplifying” regulation. Through analysis of cell size variation, we found that our cell line G_1_ distributions were most effectively modeled by a one-(growth) stage cycle, implying the dominance of a small subset of proliferative regulators. This suggests a more central role for the *RB1* subscaling observed in these cell lines.

We observed an up-regulated inflammatory response and decreased D/C ratio in larger cell lines—phenomena recently related to the onset of cell senescence (*17*). However, while larger lines appear morphologically senescent and exhibited a “senescent-like” proteomic signature (*14*, *18*), they are clearly not senescent, as they grow faster than, and proliferate at a similar rate to, smaller cells. This finding is particularly notable given the observed down-regulation of canonical pro-biosynthetic phosphorylations (for example, in the *AKT-mTOR* pathway; see fig. S5). This shows that larger cell lines maintain high growth rates despite down-regulation of anabolic pathways and decreased ribosomal mass fractions, qualities typically associated with decreased biosynthesis (*53*, *54*).

The mechanism behind how our larger cell lines avoid senescence and maintain cell growth is unclear but may relate to mechanobiological processes, given the observed up-regulation of ECM components and RAC GTPase signaling in larger cell lines. Mechanical activation of *YAP/TAZ* signaling has been observed to facilitate growth/proliferation under *MAPK* inhibition (*55*, *56*). Furthermore, cell volume has recently been tied to substrate stiffness and adherence, engaging in a feedback system with *YAP/TAZ* (*57*). Several studies suggest that actomyosin contractility during cell spreading can also reduce cell volume through the expulsion of water, concentrating cell constituents (*58*, *59*). Large cell lines may activate cytoskeletal signaling to concentrate key biosynthetic regulators and sustain growth.

Further, by interrogating the α (mitogenic signaling) and β (growth rate) parameters of our model, we found that they were best predicted by the expression of *TP53* or *CDKN1A/CCND1*. We observed that high *CDKN1A/p21* and *TP53* mass fractions were mutually exclusive in large, high growth rate cell lines. Whether the high expression of *TP53* or *CDKN1A* in large cells is critical to their proliferation or rather simply a signature of senescent-like states is not clear. One simple explanation is that increased size provokes stress and up-regulation of* TP53* and its downstream effector *CDKN1A/p21*, consistent with other studies (*14*, *17*, *18*, *60*). While in normal cells, this would lead to cell cycle arrest (*61*), activating mutations in *BRAF* and *NRAS*, combined with mutation in tumor suppressors such as *PTEN* and can result in robust mitogenic signaling and translation of *CCND1*, which may “override” *CDKN1A/p21*-mediated checkpoint activation. However, in KD*BRAF*-mutated cells, the dynamics of *TP53*-mediated up-regulation of *CDKN1A/p21* appear altered such that *CDKN1A/p21* fails to accumulate. This may allow cell cycle entry at the relatively low levels of *CCDN1* that are present in KD*BRAF* cells.

However, we cannot exclude the possibility that *TP53* or *CDKN1A/p21* may actually facilitate progression in large cells, by resolving stresses or acting at checkpoint proteins. For example, *TP53* or* CDNK1A/p21 *expression in large cells might act to slow proliferation transiently (as cells grow), allowing engagement of the DDR (*61*) or other processes that can ultimately promote cell cycle entry.

**Fig. 8.**
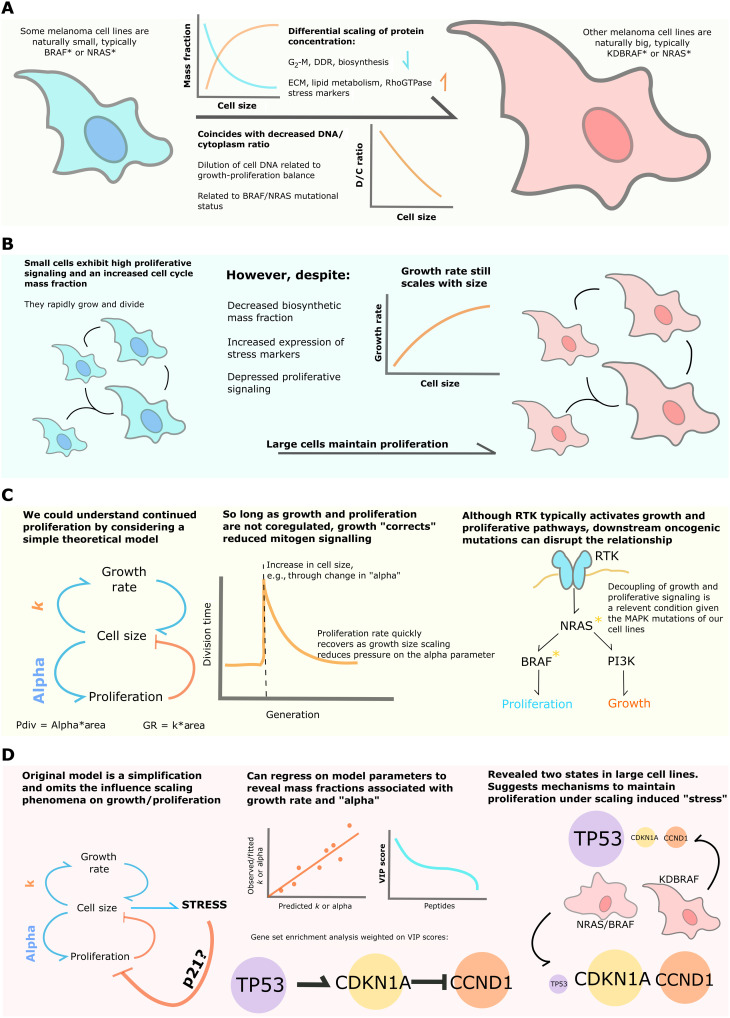
Summary of major findings. (**A**) We leveraged the size heterogeneity of *BRAF*, *NRAS*, and KD*BRAF*-*NRAS* melanomas to study how peptides, transcripts, and phosphorylation events scale with cell size and DNA-cytoplasm ratio. (**B**) The scaling relationships identified implied decreased biosynthesis and proliferative capacity in larger cell lines; unexpectedly, we found that larger cell lines exhibit higher growth rates and maintain proliferation. (**C**) We understood the proliferation of larger cell lines using a simple theoretical model. This relied on the probability of passing a cell cycle stage being related to size and a size-dependent growth rate. Given that size and growth signaling are independent, as is expected from our cell lines mutations in the *MAPK* pathway, growth provides a means to correct reduced mitogen signaling in our system. (**D**) We were aware that our model represents a simplification, and so we investigated possible molecular influences of our model parameters by constructing linear models predicting them from the proteomic expression data. Conducting GSEA on peptides most strongly contributing to the model, we identified the expression and regulation of the *TP53-CDKN1A-CCND1* axis as a major predictor of cell growth rate and α. Further examination of expressions revealed two distinct states of the system in larger cell lines.

Together, our data show that despite subscaling relationships between key biosynthetic/proliferative regulators and cell size and increased expression of stress markers, larger melanoma cell lines, with lower D/C ratios, exhibit a higher growth rate than smaller lines and maintain proliferation. Theoretical modeling suggests that proliferation may be sustained under mitogenic inhibition by decoupling growth and proliferative signaling (Fig. 8). Our work provides a generalizable, integrative framework for understanding the wiring and dynamics of signaling networks in the context of cellular morphology.

## METHODS

### Cell culture

Cell lines were maintained in standard culture conditions [Dulbecco’s modified Eagle’s medium (DMEM) + 10% fetal bovine serum (FBS); vessel; Corning Primaria 25-cm^2^ rectangular canted neck cell culture flask with vented cap; PN: 353808]. Passage was carried out using 0.25% trypsin-EDTA (Gibco) followed by centrifugation (1000 rpm, 4 min) and resuspension in complete medium. Cell counting was performed using a Countess automated cell counter with trypan blue exclusion (Thermo Fisher Scientific).

### Growth curves

Each cell line was seeded into three wells of a six-well tissue culture plate [Falcon six-well clear flat-bottom tissue culture–treated multiwell cell culture plates (Corning, PN: 353046)]. Cells were incubated in DMEM media with 10% FBS and Primocin antibiotic at 37°C and 5% carbon dioxide. Cells were imaged at 4-hour intervals using the Incucyte imaging system. Nine fields of view were imaged from each well. Images were segmented using Ilastik image segmentation software to identify individual cells. The number of cells in each field of view was calculated using CellProfiler. Growth curves were plotted using the ggplot2 library from the R programming language. Average growth rates were computed as Gr = 2/3*(Mean_Area)/(Doubling_time), where doubling time is the average time taken for cell number to double in the population.

### Immunostaining

Samples were fixed in freshly prepared 4% paraformaldehyde (PFA)/phosphate-buffered saline (PBS) for 15 min. Slides were subsequently permeabilized with 0.25% Triton/PBS for 10 min and blocked with 0.5% bovine serum albumin (BSA)/0.02% glycine/PBS for 30 min. Primary antibodies were introduced via the same solution in a 1:1000 dilution and left on for 1 hour. The slides were washed with PBS, and the same was carried out for the secondary antibodies (kept in the dark to avoid bleaching). Hoechst stain was added postsecondary (1:500) to stain DNA as was phalloidin to stain actin.

### Image acquisition and feature extraction

Image acquisition was performed using an Opera Cell:Explorer-automated spinning disk confocal microscope. Twenty fields of view were imaged in each well (PerkinElmer, PhenoPlate 384-well, black, optically clear flat-bottom, tissue culture treated, PN: 6057302). Cell segmentation was performed using Acapella software (PerkinElmer). Nuclei were segmented using the Hoechst channel (405 to 450) and cell bodies defined by the tubulin signal (568 to 603). Geometric features measured include the area of all subcellular regions; the length, width, and elongation (length/width) of the cell and nucleus; cell and nuclear roundness; and nucleus area/cytoplasm area.

### Statistical analysis of cell size

Statistical tests were carried out in the MATLAB (MathWorks) environment. Cell area data were “acosh”-transformed to induce a normal distribution of areas in each cell line and standardize the variances before ANOVA and Mann-Whitney/Wilcoxon tests. Standardization success was determined using the Shapiro-Wilks normalization test, ensuring that the data are normally distributed, and the Bartlett test, to guarantee equal variances across lines.

### FACS analysis

Cells were trypsinized and harvested into a 15-ml falcon tube for cell counting. After centrifuging the falcon at 2400 rpm for 5 min, the supernatant was discarded, and the cells were resuspended in 1-ml of 1% fetal calf serum in PBS. Three-milliliter ice cold 100% ethanol was added dropwise to the cells while slowly vortexing and left to fix overnight. The cells were then pelleted by centrifugation for 5 min at 2400 rpm and resuspended in 5 ml of PBS. They were incubated at room temperature for 20 min. After centrifuging for 7 min at 1200 rpm, the pellet was resuspended in 1 ml of propidium iodide (PI) solution through the cell strainer into a FACS tube. The PI solution was made with 1:100 PI at 5 mg/ml and 1:1000 ribonuclease A at 10 mg/ml in PBS. The cell cycle composition was measured using the BDSAria, and the data were analyzed using FlowJo. For EdU (5-ethynyl-2′-deoxyuridine) incorporation assays, cells were treated with a final concentration of 10 μM EdU before harvesting. Instead of fixing with ethanol and staining with PI, cells were resuspended in 4% PFA for 15 min at room temperature. They were then pelleted by centrifugation and PFA aspirated, followed by a wash. Five hundred microliters of the appropriate Thermo Fisher Scientific Click-iT reaction cocktail was added to each sample and incubated for 30 min in the dark, according to the manufacturer’s instructions. Cells were washed once, stained, and then transferred via a cell strainer into a FACS tube for analysis as above. Washes used 1% BSA in PBS. Staining used Hoechst (20 μg/ml) added to 0.1% Triton-X in PBS. If applicable, 10^6^ cells were seeded in Falcon T25 flasks and incubated overnight in media containing the appropriate aphidicolin concentration (total volume of 4 ml) before FACS analysis of DNA content as above.

### Proteomic sample preparation

Cell pellets were dissolved in 150 μl of lysis buffer containing 1% sodium deoxycholate, 100 mM triethylammonium bicarbonate, 10% isopropanol, 50 mM NaCl, and Halt protease and phosphatase inhibitor cocktail (100×) (Thermo Fisher Scientific, #78442) on ice with pulsed probe sonication for 15 s. Samples were boiled at 90°C for 5 min and sonicated for another 5 s. Protein concentration was measured with the Quick Start Bradford Protein Assay (Bio-Rad) according to the manufacturer’s instructions. Aliquots containing 100 μg of protein were reduced with 5 mM tris-2-carboxyethyl phosphine for 1 hour at 60°C and alkylated with 10 mM iodoacetamide for 30 min in the dark. Proteins were then digested overnight by adding trypsin at final concentration of 75 ng/μl (Pierce). The resultant peptides were labeled with the tandem mass tag (TMT)-11plex reagents (Thermo Fisher Scientific) according to the manufacturer’s instructions and were combined in equal amounts into a single tube. The combined sample was then dried with a centrifugal vacuum concentrator. Two technical replicate TMT batches from the same protein extracts were prepared to assess reproducibility. One TMT batch was fractionated offline with high-pH reversed-phase chromatography using the XBridge C18 column (2.1 mm by 150 mm, 3.5 μm, Waters) on a Dionex UltiMate 3000 high-performance liquid chromatography (HPLC) system. Mobile phase A was 0.1% ammonium hydroxide (v/v), and mobile phase B was acetonitrile and 0.1% ammonium hydroxide (v/v). The TMT-labeled peptide mixture was reconstituted in 100 μl of mobile phase A and fractionated with a gradient elution method at 0.2 ml/min as follows: for 5-min isocratic at 5% B, for 35-min gradient to 35% B, gradient to 80% B in 5 min, isocratic for 5 min, and re-equilibration to 5% B. Fractions were collected every 42 s and vacuum-dried. The second TMT replicate batch was fractionated with the Pierce High pH Reversed-Phase Peptide Fractionation Kit according to the manufacturer’s instructions.

### Phosphopeptide enrichment

Peptide fractions from the first TMT batch were reconstituted in 10 μl of 20% isopropanol and 0.5% formic acid binding solution and were loaded on 10 μl of phosphopeptide enrichment immobilized metal affinity chromatography resin (PHOS-Select Iron Affinity Gel, Sigma-Aldrich) already washed and conditioned with binding solution in custom-made filter tips fitted on Eppendorf tube caps. After 2 hours of binding at room temperature, the resin was washed three times with 40 μl of binding solution at 300 g, and the flow-through solutions were collected for total proteome analysis. Phosphopeptides were eluted three times with 70 μl of 40% acetonitrile and 400 mM ammonium hydroxide solution. Eluents and flow-through samples were vacuum-dried and stored at −20°C until the liquid chromatography–mass spectrometry (LC-MS) analysis.

### LC-MS analysis

LC-MS analysis was performed on the Dionex UltiMate UHPLC 3000 system coupled with the Orbitrap Lumos mass spectrometer (Thermo Fisher Scientific). Peptides were loaded to the Acclaim PepMap 100, 100 μm by 2 cm C18, 5 μm, 100 Ȧ trapping column at a flow rate of 10 μl/min. The sample was then subjected to a gradient elution on the Acclaim PepMap RSLC (75 μm by 50 cm, 2 μm, 100 Å) C18 capillary column at 45°C. Mobile phase A was 0.1% formic acid, and mobile phase B was 80% acetonitrile and 0.1% formic acid. The separation method at flow rate 300 nl/min was as follows: for 90 min (or 150 min for the replicate batch) gradient from 10 to 38% B, for 10 min up to 95% B, for 5 min isocratic at 95% B, reequilibration to 10% B in 5 min, for 10 min isocratic at 10% B. Precursors between 375 and 1500 mass/charge ratio were selected with mass resolution of 120 K, automatic gain control (AGC) 4 × 10^5^, and 50 ms ion trap (IT) with the top speed mode in 3 s and were isolated for collision-induced dissociation (CID) fragmentation with quadrupole isolation width 0.7 Th. Collision energy (CE) was 35% with AGC 1 × 10^4^ and IT 50 ms. MS3 quantification was obtained with Higher-energy C-trap dissociation (HCD) fragmentation of the top five most abundant CID fragments isolated with Synchronous Precursor Selection. Quadrupole isolation width was 0.7 Th, CE 65%, AGC 1 × 10^5^, and 105 ms IT. The HCD MS3 spectra were acquired for the mass range 100 to 500 with 50 K resolution. Targeted precursors were dynamically excluded for further isolation and activation for 45 s with 7 parts per million (ppm) mass tolerance. Phosphopeptide samples were analyzed with an HCD method at the MS2 level with CE 38%, AGC 1 × 10^5^ and max IT 105 ms.

### Database search and protein quantification

The SequestHT search engine was used to analyze the acquired mass spectra in Proteome Discoverer 2.2 (Thermo Fisher Scientific) for protein identification and quantification. Precursor mass tolerance was 20 ppm, and fragment ion mass tolerance was 0.5 Da for the CID and 0.02 Da for the HCD spectra. Spectra were searched for fully tryptic peptides with maximum of two miss-cleavages. TMT6plex at N terminus/K and carbamidomethyl at C were defined as static modifications. Dynamic modifications included oxidation of M and deamidation of N/Q. Dynamic phosphorylation of S/T/Y was included for the phospho-enriched samples. Peptide confidence was estimated with the percolator node. Peptide FDR was set at 0.01, and validation was based on *q* value and decoy database search. Spectra were searched against reviewed UniProt mouse protein entries. The reporter ion quantifier node included a TMT 11plex quantification method with an integration window tolerance of 15 ppm and integration method based on the most confident centroid peak at the MS3 or MS2 level. Only unique peptides were used for quantification, considering protein groups for peptide uniqueness. Peptides with average reporter signal-to-noise of >3 were used for quantification.

### Proteomic/transcriptomic analysis

Peptide abundances were scaled relative to other detected peptides in the sample such that they reflect abundance/total protein mass. The expression of each peptide was correlated to average cell line area to derive a correlation coefficient, *R*, and a fold change was calculated between cells lying above or below the mean. Peptides scoring Fc > 1.5 or <0.66 and |*R*| > 0.55 were taken as hit super/subscaling peptides. The Fc threshold of 1.5 stems from a power analysis: An Fc of 1.5 is the minimal detectable difference between groups we can detect by a *t* test between small and large cell line clusters (centered about “100 scaled units”) when accepting a true-positive rate of 0.95 [where *n* = 5 and 6, cluster SDs were taken to be 20 scaled units (the SD of expressions within a class)]. The fold change was defined as

$$\mathrm{Fc}=\frac{M(A)}{A},\mathrm{when}\;\frac{1}{2}[A+M(A)]=100$$ (9)

where “*A*” denotes the mean abundance of the peptide in cluster 1, and *M*(*A*) denotes the minimal value of the mean of cluster 2 for there to be a detectable difference in mean as above. The “100” stems from all peptides being scaled across line to have a mean value of 100. This value was calculated using the “samplesizepwr” function in MATLAB (MathWorks) statistics and machine learning toolbox. Transcripts are treated identically to peptides.

### Network analysis

High-scoring proteins are taken forward and entered into STRING (*41*) to screen for interactions within the hits. Accepted interactions were those identified experimentally or identified in previous coexpression studies and achieved a confidence value > 0.4. This network was then exported to Cytoscape (*62*) for ontological analysis via the SAFE (*42*) tool. All “biological process” annotations for each node in the network were derived from Geneontology.org’s downloadable database (*43*–*46*). A binary matrix was constructed; each node (row) would receive either a 1 or 0 in each column (annotation) depending on whether the node was associated with the label. This was then entered into the SAFE Cytoscape plugin, where we used default settings besides a percentile threshold of 10 and minimum neighborhood size of 5 (data S5). A second binary matrix was then constructed now with an annotation set reflecting whether the node was an expression or phosphorylation hit in big or small cells. The same settings were used for SAFE.

### RNA extraction, quality control, and RNA sequencing

RNA from 11 cell lines was extracted using the RNeasy Mini Kit (Qiagen, #74104) according to the manufacturer’s protocol. The evaluation of the isolated RNA integrity and quantity was carried out by the Agilent TapeStation system using an RNA ScreenTape (Agilent Technologies, #5067-5576).

For the mRNA library preparation, 4000 ng of total RNA was treated with TurboDNase to remove genomic DNA contamination, (Invitrogen, #AM1907). PolyA RNA was selected from 1000 ng of the purified RNA using NEBNext mRNA magnetic isolation module [New England BioLabs (NEB), #E7490] following the manufacturer’s directions. From the resulting mRNA, strand-specific libraries were created using the NEBNext Ultra II Directional RNA Library Prep Kit for Illumina (NEB, #E7760). Final libraries were quantified using quantitative polymerase chain reaction and clustered at a molarity of 300 pM. Sequencing was performed on an Illumina NovaSeq 6000 (Illumina) using paired end ×100 cycles v1.0 chemistry, to achieve coverage of 25 million reads per sample.

### Model algorithm

The initial cell area distribution is considered a delta function centered on *k/*α (the expected mean of the distribution). Every generation, the area distribution is convolved with the mass-gain distribution, computed by performing an inverse Fourier transform on the product of the two distributions respective Fourier transforms. This produces the division area distribution, Ad(A), which must be transformed to Ad(2A) to capture the effects of cell division. We perform this by setting Ab(Ax) = Ad(Ai) + Ad(Ai + 1), where “I” = *x*_n_ − *x*_n-1_ for all *x*, where Ab denotes the birth size distribution. This is then convolved with the gain distribution as before to generate the next division distribution and so on until a desired number of generations has been reached.

### Numerical simulation

An initial population of 1000 cells was assigned an α and β value and a random initial area. At each time step, the division probability for each cell is calculated, according to *P* = α*A*_div_, and a random number, “*r*,” is drawn from a flat distribution. Should *r* be less than the division probability of a cell, the cell divides symmetrically in two, adding a new cell to the population with half the size of the mother, and halving the mother size. If *r* is greater than the division probability, then the cell size increases according to β = *kA*_div_. This system continues until a final cell count of 20,000 was achieved.

### Model fitting procedure

Initially, α values were exhaustively tested (β is determined from the proliferation and area measurements on a per cell line basis). For each, we calculated the Kullbeck-Liebler divergence between the experimental and simulated data

$$D_{\mathrm{KL}}(P\|Q)=\sum_{x\in X}P(x)\log_{10}\left[\frac{P(x)}{Q(x)}\right]$$ (10)

Having identified approximate minima from the low-resolution parameter screen, we used the values defining this region as an initial state for a stochastic gradient descent minimizing along the gradient

$$\frac{dD_{\mathrm{KL}}(P\|Q)}{dp}=4[P(x)-Q(x)]Q(x)$$ (11)

Model fitting was conducted within the commercial MATLAB (MathWorks) software’s machine learning toolbox.

### Partial least squares regression

Regression analyses were conducted with the MATLAB (MathWorks) environment using the plsregress function from the machine learning toolbox. Partial least square regression was selected as the method to help mitigate the influence of colinearity in the predictor dataset. Model components were selected through fivefold cross validation using the elbow method on the mean square error as a function of component number. All peptides were mean-centralized before model construction. Fit quality was assessed through the *r*-squared metric.

### Feature importance to PLSR models

The influence a feature has on a model was estimated through VIP scores calculated as

$${\mathrm{VIP}}_{j}=\sqrt{\frac{\sum_{f=1}^{F}w_{jf}^{2}\cdot SSY_{f}\cdot J}{{SSY}_{\mathrm{total}}\cdot F}}$$ (12)

*wjf* is the weight value for *j* variable and *f* component, and SSY*_f_* is the sum of squares of explained variance for the *f*th component and *J* number of *X* variables. SSY_total_ is the total sum of squares explained of the dependent variable, and *F* is the total number of components. Features with a VIP score greater than 1 were taken as major drivers of the model. VIP scores of peptides with negative weights in the first PLSR component were made negative in preparation for enrichment analysis.

### Gene set enrichment analysis

GSEA was conducted using the “WebGestalt” web application on our ranked list of peptides (VIP score defined the rank) (*63*). We used the “network” and “PPI_BIOGRID” enrichment categories to identify enriched subnetworks in the high- and low-scoring peptides. Parameters used were as follows: minimum IDs per category = 5; maximum = 10,000; permutations = 1000. Enrichments with an FDR < 0.05 were taken as hit subnetworks. We conducted an additional over-enrichment analysis using the “GOrilla” webtool comparing hit peptides, as defined in the “Proteomic/transcriptomic analysis” section, against all detected species. Default parameters were used.

## Supplementary Materials

### This PDF file includes:

Analysis of gene and theme overlap of size-scaling factors between datasets Derivation of the proliferation time and size gain distributions Deriving the moments of the cell size distribution Figs. S1 to S6

## Notes

View/request a protocol for this paper from *Bio-protocol*.
