# Supplementary material for: Characterization of proteome-size scaling by integrative omics reveals mechanisms of proliferation control in cancer
Source: Sci Adv. 2023 Jan 25;9(4):eadd0636. doi: 10.1126/sciadv.add0636 (PMC9876555; doi:10.1126/sciadv.add0636)
Supplement: Supplementary file 1 — Analysis of gene and theme overlap of size-scaling factors between datasets Derivation of the proliferation time and size gain distributions Deriving the moments of the cell size distribution Figs. S1 to S6 [file sciadv.add0636_sm.pdf]

Science Advances
AAAS

Supplementary Materials for

Characterization of proteome-size scaling by integrative omics reveals mechanisms of proliferation control in cancer

Ian Jones *et al.*

Corresponding author: Ian Jones, ian.jones@icr.ac.uk; Chris Bakal, chris.bakal@icr.ac.uk

Sci. Adv. **9**, eadd0636 (2023)
DOI: 10.1126/sciadv.add0636

The PDF file includes:

Analysis of gene and theme overlap of size-scaling factors between datasets
Derivation of the proliferation time and size gain distributions
Deriving the moments of the cell size distribution
Figs. S1 to S6
Legends for data files S1 to S7

Other Supplementary Material for this manuscript includes the following:

Data files S1 to S7

Supplemental Information:

Analysis of gene and theme overlap of size-scaling factors between datasets:

We investigated which ontological themes were enriched in both analyses finding that peptides pertaining to cell cycle, DNA repair, and division processes remained enriched in smaller cell lines (eg; 'DNA repair', 'Cell cycle process', 'Cytokinesis', 80% A1-A2, 16 % A2-A1 indicating 80% of themes enriched in the first analysis match the second and 16% detected in the second match the first) whilst lipid and carbohydrate metabolic peptides (eg; 'Lipid metabolic process', 'Carbohydrate derivative metabolic process', 'Sterol metabolic process', 30% A1-A2, 21% A2-A1) are consistently enriched in larger cell lines. Due to the lack of agreement, the enrichment of ECM components in larger cell lines detected in the prior analysis may reflect an upregulation or overexpression rather than a scaling relationship. Enacting the same analysis for the phosphorylation data, we note excellent agreement between analyses (63% A1 -A2, 60% A2-A1) for small cell lines, with both enriching for cell cycle and biosynthetic processes (eg; regulation of cellular biosynthetic process, mitotic cell cycle, DNA replication). Larger cell lines exhibited much weaker agreement (9% A1-A2, 30% A2-A1) but both analyses revealed enrichment of cytoskeletal and GTPase regulatory phosphorylations (eg; Regulation of GTPase activity, 'Cell junction assembly', 'Actin filament based process') (**SF3**).

Investigating the overlap of individual genes, we note a particularly strong overlap between analyses for phosphopeptides enriched in smaller cell lines (36% A1-A2, 30% A2-A1). Phosphopeptides enriched in larger cell lines show a more modest overlap (16% A1-A2, 14% A2-A1) like that observed in peptide expressions for smaller cell lines (15% A1-A2, 27% A2-A1). Peptide expressions in larger cell lines exhibit the weakest overlap (5% A1-A2, 8% A2-A1) (**SF3**). Screening for interactions between overlapping genes we observe a set of 21 physically interacting genes centred on BRCA1 enriched in smaller cell lines. As a 'hit' in two separate scaling analyses, these data indicate that the BRCA1 complex scales with cell size (**SF3**).

These data corroborate our previous analysis, strengthening the claim that G2/M and DNA repair processes define smaller melanoma cell lines, (with associated peptides sub-scaling with cell size), whilst cytoskeletal organisation and the rewiring of lipid

metabolism define larger cell lines (peptides super-scaling with size). Interestingly we recover a large, BRCA1 complex in both analyses, implicating the complex in size-dependent phenomena.

Derivation of the proliferation time and size gain distributions:

We are interested in the waiting time distribution before the first successful event. The probability to fail a division is:

$$P_{fail} = 1 - P_{div} \quad equ.S1$$

For a cell to have not divided by a given time point, it must have failed to divide at every prior time point. The probability of successive failures occurring at a given time is equal to:

$$F(t) = \left(P_{fail}\right)^t = (1 - \alpha A_{div})^t \quad equ.S2$$

Where ‘t’ is time since the last division. The probability of having divided by a given ‘t’ is the probability that the cell has not failed at every prior step:

$$C(t) = 1 - (1 - \alpha A_{div})^t \quad equ.S3$$

The probability distribution follows as:

$$P(t) = \frac{d}{dt}[1 - (1 - \alpha A_{div})^t] = -(1 - \alpha A_{div})^t \ln(1 - \alpha A_{div}) \quad equ.S4$$

$$P(t) = \lambda e^{-\lambda t} \quad , \quad \lambda = -\ln(1 - \alpha A_{div}) \quad equ.S5$$

We may extract the expected gained mass by scaling the time by ln(2)/(dt/dA). The ln(2) factor accounts for a division event having happened any time in the interval 0-t.

$$C(A(t)) = 1 - (1 - \alpha A_{div})^{\frac{A(t)}{\ln(2) A_{div} k}} \quad equ.S6$$

$$P(A(t)) = -(1 - \alpha A_{div})^{\frac{A(t)}{\ln(2) A_{div} k}} \left[\left(\frac{1}{\ln(2)\, k A_{div}}\right) \ln(1 - \alpha A_{div})\right] \quad equ.S7$$

$$P(A(t)) = \lambda e^{-\lambda A(t)} \quad , \quad \lambda = -\frac{1}{\ln(2)\, A_{div} k} \ln(1 - \varphi A_{div}) \quad equ.S8$$

With a mean of known form given as $1/\lambda$:

$$\langle P(t) \rangle = \frac{-\ln(2) k A_{div}}{\ln(1 - \alpha A_{div})} \quad equ.S9$$

We can see that this result constitutes an adder –type system when expressing the expected area gain as a Laurent series about α = 0 (fitted values never exceed 1X10^-5) (F6B/C):

$$\frac{-\ln(2)kA_{div}}{ln\,(1-\ \alpha A_{div})} = \frac{\ln(2)k}{\alpha} - \frac{kA_{div}}{2} - \frac{1}{12}{A_{div}}^2\alpha k - \frac{1}{24}{A_{div}}^3\alpha^2 k \ldots \qquad equ.S10$$

It is clear that the mean area gain is approximately constant, as the first term dominates the expression by virtue of alpha ≈ 0. Thus, a constant average mass is added each cycle, despite the area gain distribution itself being dependent on division size.

Deriving the moments of the cell size distribution:

Starting with an initial size distribution, F(A), and size gain distribution, G(A), we may define the expected size distribution up to the first division, H(A) as:

$$F(A) * G(A) = H(A) \quad equ.S11$$

On division, the value of cell size is considered to halve. Thus, the birth size distribution is given as:

$$F(2A) * G(2A) = H(2A) = B(A) \quad equ.S12$$

Where the inclusion of 2A has mapped the probability of A to half its value, thereby simulating a division event. This is then convolved with G(A) again for the next division cycle, and so on:

$$\left[[F(2^nA) * G(2^nA)] * G(2^{n-1}A)\right] \ldots * G(A) \ = H(A) \quad equ.S13$$

Where n denotes the number of divisions. Note that as n increases, the influence of the initial size distribution on the total convolution decreases as $F(2^nA)$ has non-zeros values only at extremely low sizes as n increases. Indeed, we can approximate the above as:

$$P_{Div}(A) = \ \left[[F(2^nA) * G(2^nA)] * G(2^{n-1}A)\right] \ldots * G(A)$$

$$\approx G(A) * G(2A) * \ldots G(2^nA) \quad equ.S14$$

G(A) has been shown to be an exponential distribution. Convolution of n exponential functions with different scale parameters results in a hypo-exponential function with mean equal to the sum of the means of all participating distributions:

$$\langle P_{Div}(A)\rangle = \frac{1}{\lambda} + \frac{1}{2\lambda} + \frac{1}{4\lambda} + \cdots \frac{1}{2^n\lambda} \quad equ.S15$$

The sum can be written as:

$$\frac{1}{\lambda} + \frac{1}{2\lambda} + \frac{1}{4\lambda} + \cdots \frac{1}{2^n\lambda} = \frac{1}{\lambda}\left(1 + \frac{1}{2} + \frac{1}{4} + \cdots \frac{1}{2^n}\right) = \frac{2}{\lambda} \quad equ.S16$$

Indicating that the distribution tends toward a constant mean. The corresponding variance is similarly given as:

$$\langle\langle P_{Div}(A)\rangle\rangle = \frac{1}{(\lambda)^2} + \frac{1}{(2\lambda)^2} + \frac{1}{(4\lambda)^2} \cdots \frac{1}{(2^n\lambda)^2} = \frac{1}{(\lambda)^2}\left(1 + \frac{1}{4} + \frac{1}{16} + \cdots \frac{1}{2^{2n}}\right)$$
$$= \frac{4}{3(\lambda)^2} \; equ.S17$$

Yielding a constant coefficient of variation:

$$CV = \frac{\sqrt{\frac{4}{3(\lambda)^2}}}{\frac{2}{\lambda}} = \frac{1}{\sqrt{3}} \approx 0.5774 \quad equ.S18$$

These results may be trivially adjusted to account for ‘x’ identical events governing division. Indeed, G(A) is merely transformed from a constant exponential distribution to a constant Erlang distribution of shape factor ‘x’ and rate parameter 1/x k/a. This stems from G(A) being generated from the convolution of ‘x’ exponentially distributed gain variables corresponding to the area gain in each cycle stage each with mean 1/(x) k/a. As is the case for the hypoexponential, Erlang distributions have means and variance equal to the sum of those of the participating distributions allowing us to easily modify equ.S15/S17:

$$\langle P_{Div}(A,x)\rangle = \frac{x}{\lambda} + \frac{x}{2\lambda} + \frac{x}{4\lambda} + \cdots \frac{x}{\lambda 2^n} = \frac{x}{\lambda}\left(1 + \frac{1}{2} + \frac{1}{4} + \cdots \frac{1}{2^n}\right) = \frac{2x}{\lambda}$$

$$\langle\langle P_{Div}(A,x)\rangle\rangle = \frac{x}{(\lambda)^2} + \frac{x}{(2\lambda)^2} + \frac{x}{(4\lambda)^2} \cdots \frac{x}{(2^n\lambda)^2} = \frac{x}{(\lambda)^2}\left(1 + \frac{1}{4} + \frac{1}{16} \cdots\right) = \frac{4x}{3(\lambda)^2}$$

$$CV(x) = \frac{\sqrt{\frac{4x}{3(\lambda)^2}}}{\frac{2x}{\lambda}} = \frac{\sqrt{x}}{x\sqrt{3}} = \frac{1}{\sqrt{3x}} \quad equ.S19$$

Equ.S18 tells us that from the coefficient of variation, we may estimate the number of stages needed to effectively model the cell size distributions. This relationship is similar to that obtained recently (Nieto et al., 2020) where (CV)^2 was found to be proportional to one over the number of modelled cell cycle stages. Importantly, given a single value of the $'\alpha'$ or 'k' parameters, this is entirely independent of $'\alpha'$ or 'k' facilitating simple calculation of the required 'x':

$$x = \frac{1}{3(CV)^2} \quad equ.S20$$

Supplemental Figure 1: Representative images of the primary panel of cell lines

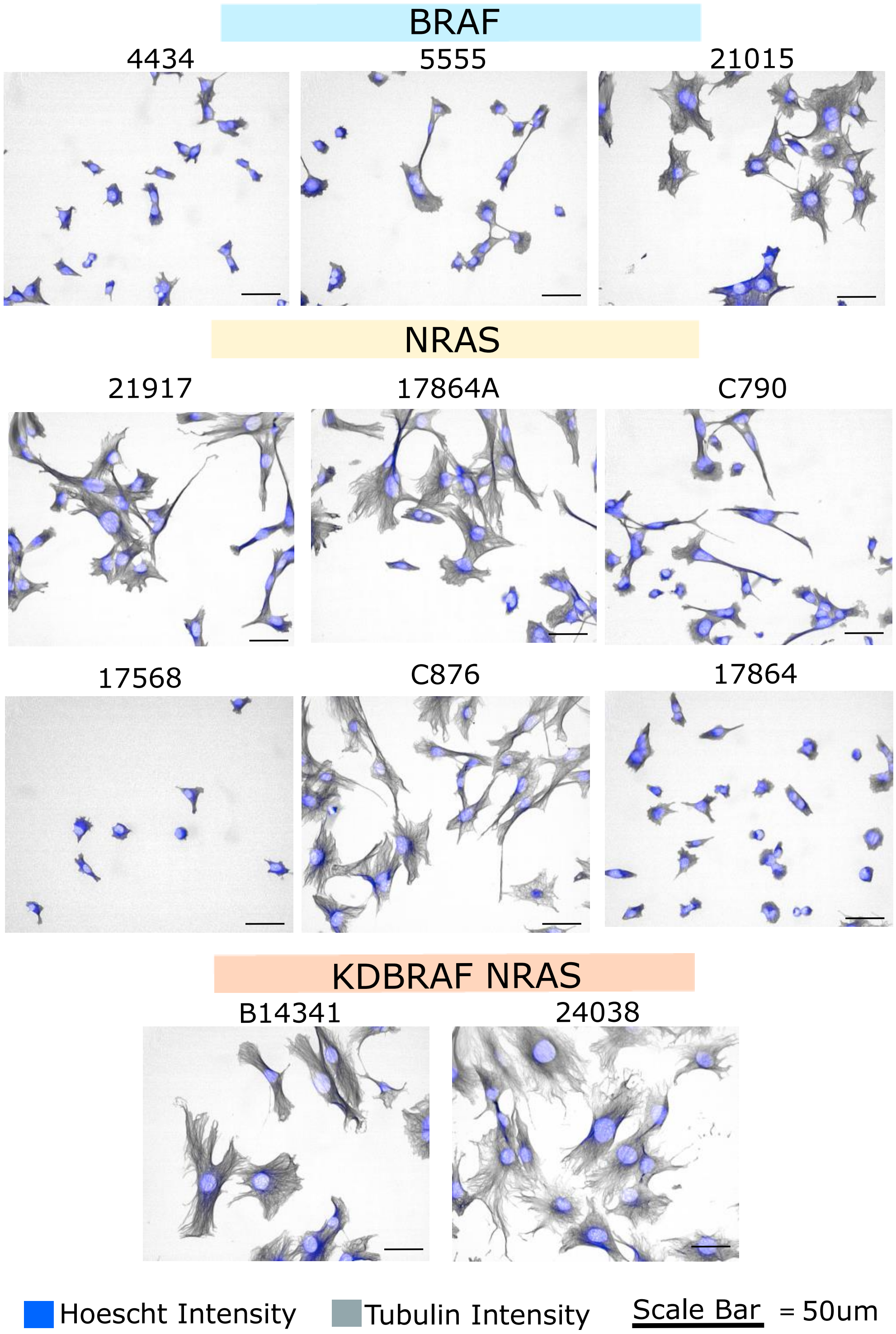

Supplemental Figure 1: Images of the cell lines: A) Representative images from the 11 cell lines. In blue is the Hoechst intensity, and grey, the tubulin intensity. All images were taken at 20X magnification using an Opera Cell: Explorer-automated spinning disk confocal microscope. Images have been auto-adjusted to optimise contrast within the acapella environment (PerkinElmer)

Supplemental figure 2: Quantification of cell DNA content via FACs analysis

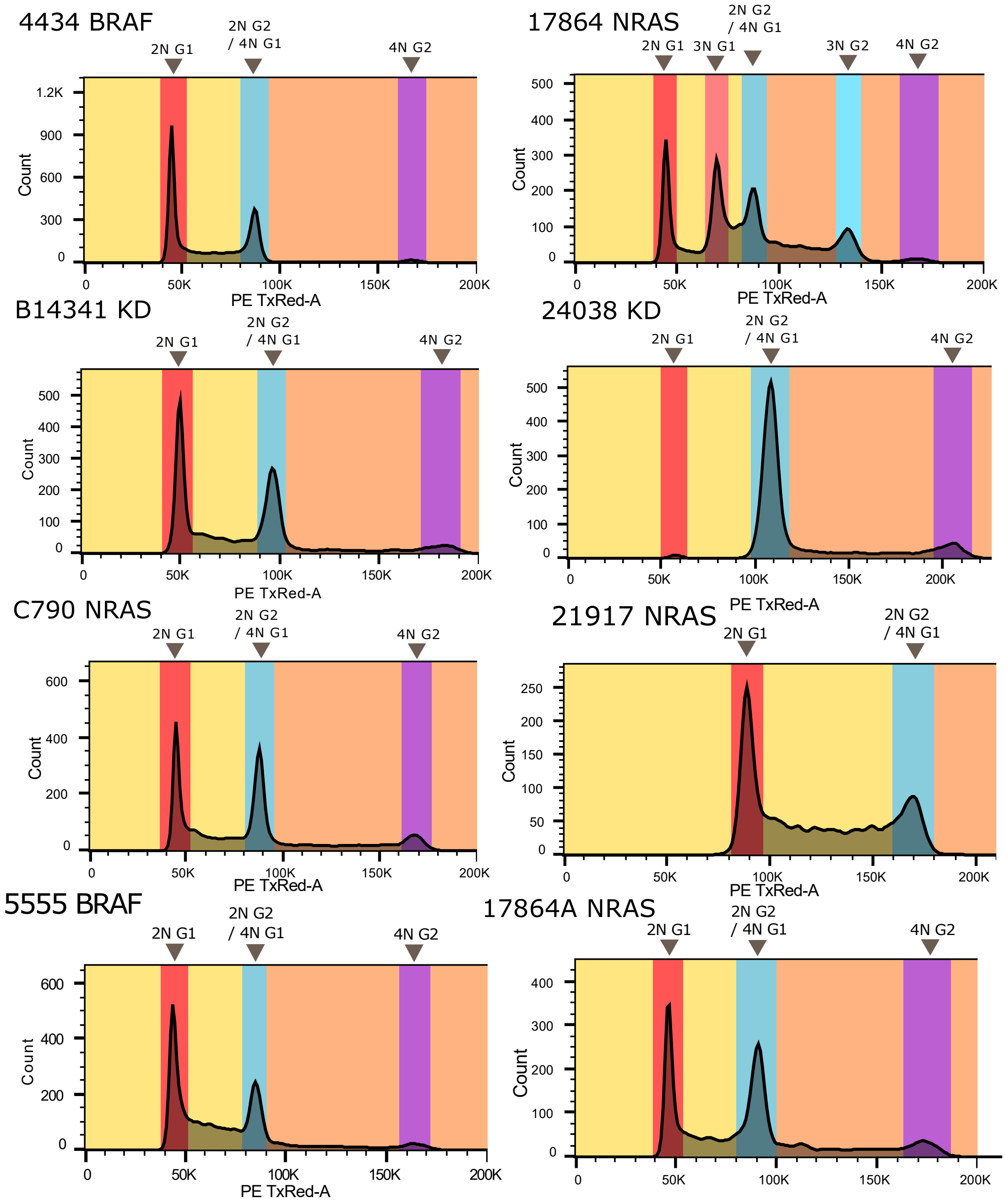

Supplemental Figure 2: FACs Analysis: A) Quantification of cell DNA through FACs analysis. Many of the cell lines, both large and small, exhibit a small polyploid population.

Supplemental figure 3: Measurement of cell growth rate

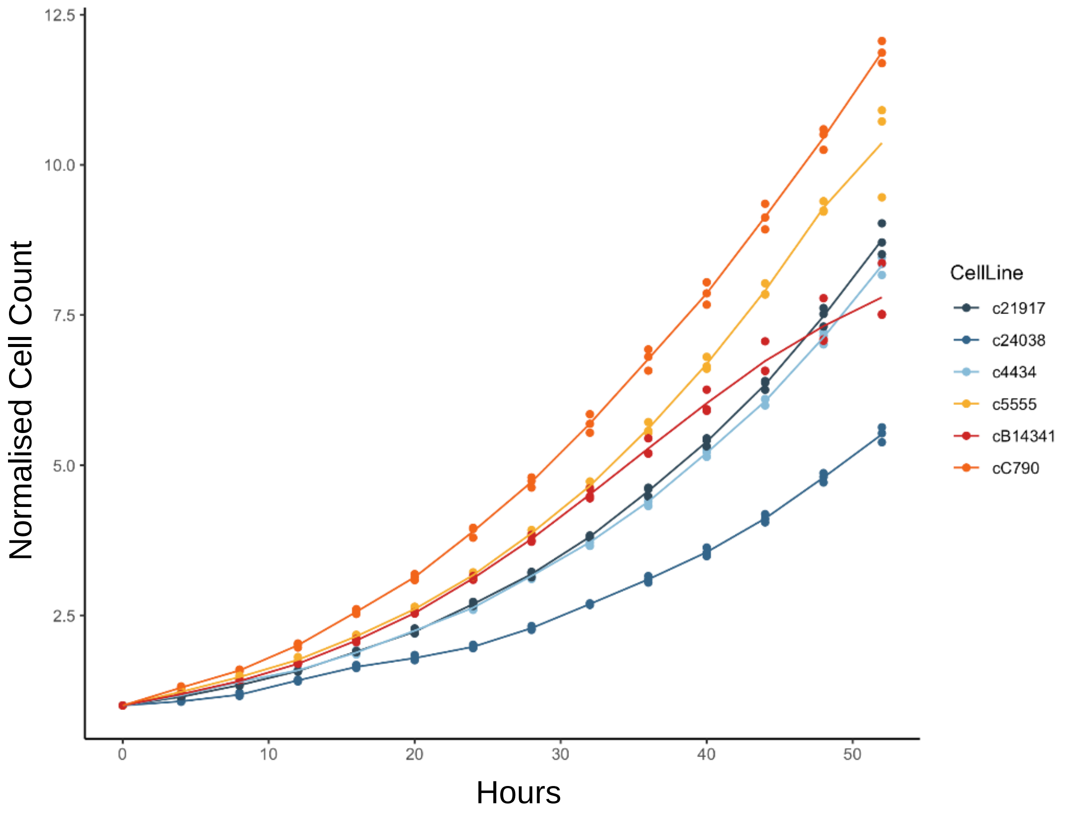

Supplemental Figure 3: Cell growth data: Cell population growth curves for a subset of the investigated lines. Cell density is normalised relative to the starting confluence of the culture. Note that a large line, B14341, shows a comparable doubling time to smaller line, 5555.

Supplemental figure 4: Validation of size controllers in an independent panel of melanoma cell lines

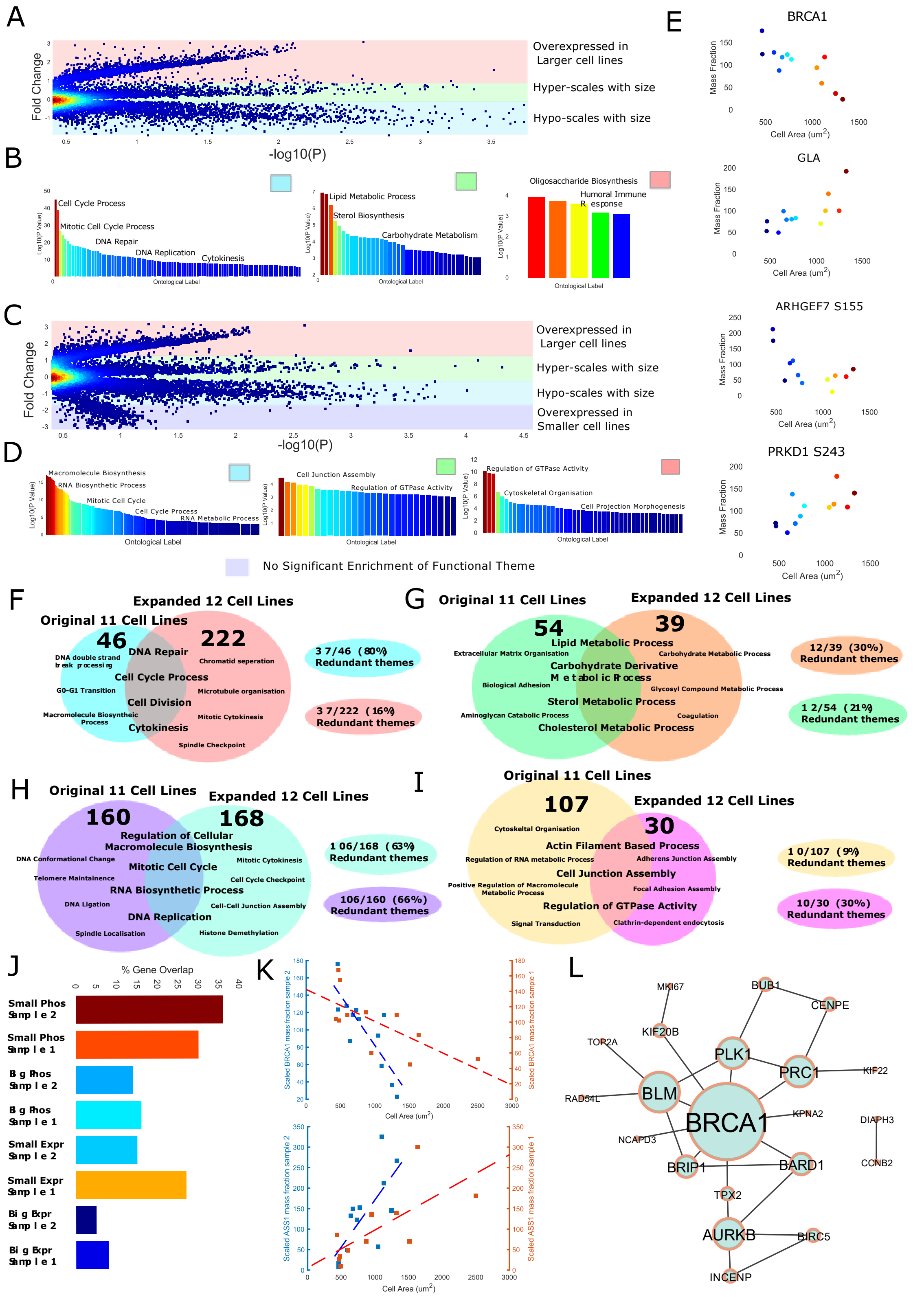

Supplemental figure 4: Validation of size controllers in an independent panel of melanoma cell lines: A) Volcano plot relating the 'fold change' across the small and large cell lines (defined as either side of the mean size) to the significance of the correlation between cell size and peptide expression. B) Themes enriched in each region of 'A' as denoted by the colour of the box in the top right of each panel. From left to right; hypo-scales with size, hyper scales with size, over expressed in large cells (enrichments significant to at least P<1X10^-3). Significance constraints for this analysis are determined identically to the original analysis C) Volcano plot relating the 'fold change' across the small and large cell lines (defined as either side of the mean size) to the significance of the correlation between cell size and phosphopeptide expression. D) Themes enriched in each region of 'C' as denoted by the colour of the box in the top right of each panel. From left to right; hypo-scales with size, hyper scales with size, over expressed in large cells (bottom = overexpressed in small cells). E) Example hits from each analysis, the top half the peptide expression analysis, the bottom, the phosphopeptide expression analysis. F-I) Venn diagrams depicting the overlap of themes enriched across both sets of cell lines. Top left = peptide expression in small lines, top right = peptide expression in big lines, bottom left = phosphopeptide expression in small lines, bottom right, phosphopeptide expression in big lines. J) Percent overlap between analyses at the gene level. K) Example genes that are hits across both analyses, the top panel shows BRCA1 peptide expression and the bottom ASS1 peptide expression. L) A set of interacting peptides derived from the overlapping list of hit genes enriched in smaller cell lines centred on BRCA1.

Supplemental figure 5: Growth signalling across cell lines

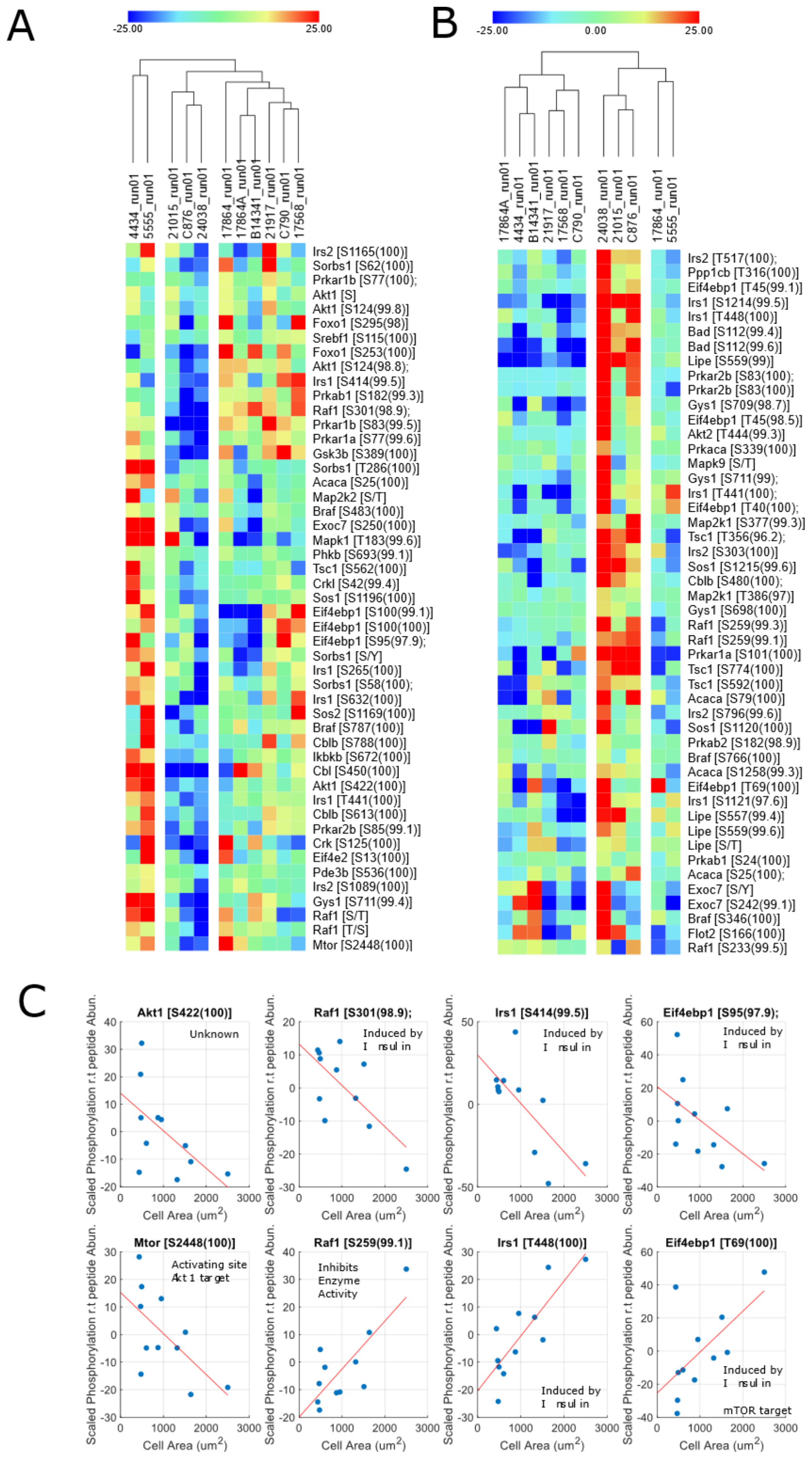

Supplemental Figure 5: Growth signalling across cell lines: A) subset of phosphopeptides that negatively correlate with cell size pertaining to the 'mTOR signalling' KEGG pathway. B) As in 'A'**,** but showing elements that positively correlate with cell size. C) Example correlation between mTOR signalling phosphorylations and cell size.

Supplemental Figure 6: Cytoskeletal phosphorylation across cell lines

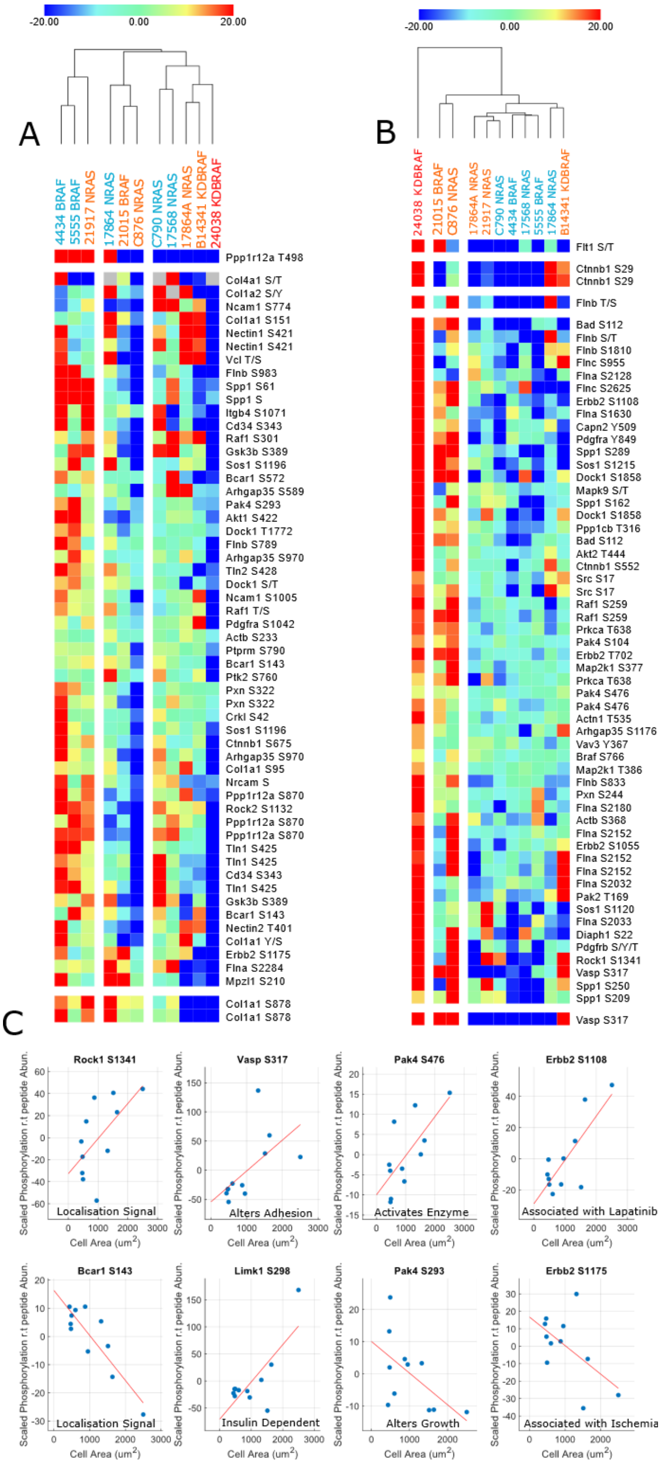

Supplemental Figure 6: Cytoskeletal phosphorylation across cell lines: A) subset of phosphopeptides that negatively correlate with cell size pertaining to the 'cytoskeleton' and 'adhesion' KEGG pathways. B) As in 'A'**,** but showing elements that positively correlate with cell size. C) Example correlation between cytoskeletal phosphorylations and cell size.

Guide to supplemental data files:

SD1: single cell morphological data for the original 11 cell lines.

'SER' are texture features reflecting the distribution of intensity of the relevant molecule. Exp4Cam3 and Exp2Cam2 refer to the tubulin signal, Exp3Cam2 the DNA signal.

SD2: processed (phospho)proteomic data for the original 11 cell lines, raw datafiles available on the PRIDE database as in text.

Pg1/4:

'Normalised' refers to detected counts for each protein divided by the total counts detected for all proteins in the cell line (and scaled)

'Scaled' refers to normalised values scaled across cell lines such that the mean normalised expression across lines equals '100' units.

Phospho-scaled:

First green rows = expression (scaled), second mass corrected (scaled) phosphopeptide expressions

Yellow = scaled phosphopeptide expressions

Other:

All expression data on other pages is 'scaled'

SD3: ontological enrichments detailed throughout the manuscript

'expanded' refers to the additional 12 cell lines used in the validation dataset

SD4: known effects and causative kinases of hit phosphorylation sites identified in this analysis

SD5: processed transcriptomic data for the original 11 lines used in this analysis

SD6: processed (phospho)proteomic data for the additional validating set of 12 cell lines

SD7: cell areas of the expanded set of 12 cell lines.
